# The Antibiotic Dosage of Fastest Resistance Evolution: Gene Amplifications Underpinning the Inverted-U

**DOI:** 10.1093/molbev/msab025

**Published:** 2021-03-08

**Authors:** Carlos Reding, Pablo Catalán, Gunther Jansen, Tobias Bergmiller, Emily Wood, Phillip Rosenstiel, Hinrich Schulenburg, Ivana Gudelj, Robert Beardmore

**Affiliations:** 1 Biosciences, College of Life and Environmental Sciences, University of Exeter, Exeter, United Kingdom; 2 Grupo Interdisciplinar de Sistemas Complejos (GISC), Departamento de Matemáticas, Universidad Carlos III, Madrid, Spain; 3 Molecular Health GmbH, Heidelberg, Germany; 4 Institute of Clinical Molecular Biology (IKMB), CAU Kiel, Kiel, Germany; 5 Evolutionary Ecology and Genetics, Zoological Institute, CAU Kiel, Kiel, Germany

**Keywords:** microbial evolution, antibiotic resistance, selection for resistance, efflux pump AcrAB-TolC, genomic amplification, prophage

## Abstract

To determine the dosage at which antibiotic resistance evolution is most rapid, we treated *Escherichia coli* in vitro, deploying the antibiotic erythromycin at dosages ranging from zero to high. Adaptation was fastest just below erythromycin’s minimal inhibitory concentration (MIC) and genotype-phenotype correlations determined from whole genome sequencing revealed the molecular basis: simultaneous selection for copy number variation in three resistance mechanisms which exhibited an “inverted-U” pattern of dose-dependence, as did several insertion sequences and an integron. Many genes did not conform to this pattern, however, reflecting changes in selection as dose increased: putative media adaptation polymorphisms at zero antibiotic dosage gave way to drug target (ribosomal RNA operon) amplification at mid dosages whereas prophage-mediated drug efflux amplifications dominated at the highest dosages. All treatments exhibited *E. coli* increases in the copy number of efflux operons *acrAB* and *emrE* at rates that correlated with increases in population density. For strains where the inverted-U was no longer observed following the genetic manipulation of *acrAB*, it could be recovered by prolonging the antibiotic treatment at subMIC dosages.

## Introduction

We treat the bacterium *Escherichia coli* at different antibiotic dosages in vitro to ascertain which one supports the most rapid resistance adaptation for a variety of genotypic and phenotypic statistics. No study addresses this question directly, to the best of our knowledge, although the literature makes relevant predictions. The mutant selection window (MSW) hypothesis, for example, predicts that resistant mutants should be detected above the minimal inhibitory concentration (MIC) of the drug-treated bacterium (Drlica 2003; Zhu et al. 2012; Pan et al. 2017; Alieva et al. 2018). Competition experiments and population genetics theory (Gullberg et al. 2011; Liu et al. 2011; Day et al. 2015; Day and Read 2016), however, indicate mutants with reduced drug susceptibility could arise, and experience positive selection, from low dosages upwards. We therefore propagate populations at sub and super-MIC dosages and seek the most rapidly adapting lineages in treatment assays described below.

The so-called inverted-U is predicted to appear in the resulting data (Tam et al. 2007; Drusano et al. 2009; Kouyos et al. 2014; Day and Read 2016), based on the following idea. Selection for resistance should increase with dose (Ankomah and Levin 2014) but, due to a declining population size, if we set aside relationships between antibiotic stresses and DNA damage (Roth 2011; Maharjan and Ferenci 2015; Long et al. 2016; Sharma et al. 2017), the supply of mutants could decrease with increasing dose. Therefore, if one were to regress dose against a quantitative measure of resistance adaptation, an inverted-U should appear where its peak marks the dose of most rapid adaptation. MSW reasoning predicts this peak should lie above the bacterium’s MIC, although population genetics theory (Day et al. 2015) indicates this is not “technically correct,” an observation that accords with recent in vitro and in vivo tests of MSW theory (Pan et al. 2017). Despite the disagreement, the inverted-U is said to be (Day and Read 2016) “*arguably the single-most robust finding in all of the empirical literature*.” Our data address both this technical disagreement and the robustness of the inverted-U.

To obtain those data, we treat *E. coli* with the antibiotic erythromycin and quantify the dose-dependent rates at which resistance mechanisms are enriched in genomic and phenotypic data. If inverted-Us are absent, or if they peak below the MIC, that would not support the MSW concept. However, treatment duration must be an important parameter mediating the absence of an inverted-U and we therefore study aspects of how both treatment duration and *E. coli* resistance genetics can affect the inverted-U. Simple theory indicates inverted-Us need not arise if the antibiotic is very effective at suppressing growth or if mutations are too rare, in which case the lowest dosages could support the most rapid adaptation. As gene amplifications typically have high mutation rates (Sandegren and Andersson 2009; Roth 2011; Tomanek et al. 2020), peaks of an inverted-U could, but need not (Langevin et al. 2020), coincide with the genomic amplification of resistance mechanisms, even though MSW theory was not originally motivated by amplifications.

Erythromycin is deployed clinically against Gram negative bacteria but it is not used to treat *E. coli*, although it likely encounters erythromycin as an unintended side-effect. So whereas our study lacks a clinical context, erythromycin is helpful for quantifying the evolutionary basis of the inverted-U because it is a substrate of the efflux pump AcrAB-TolC found in the genome of the *E. coli* (George and Levy 1983; Pena-Miller et al. 2014; Bergmiller et al. 2017). Moreover, *E. coli* is known to exhibit *acrAB* amplification mutants with decreased sensitivity to erythromycin that yield evolutionary genomic data from short-term treatments (Laehnemann et al. 2014; Pena-Miller et al. 2014). Amplification of the *acrAB* operon therefore provides a signal of genomic change that can be quantified from deep sequencing data of short-term erythromycin treatments.

When challenging *E. coli* at as many dosages as practicable, we are interested in the “nonlinear geometry” that appears when regressing a population phenotype, like population density, against dose. As population level phenotypes are selected through mechanisms expressed at the genetic level, we also seek inverted-U data for single genes by estimating the dose-dependence of selection for novel polymorphisms. An inverted-U occurs when the resulting regressions have a single peak marking the dose of most rapid adaptation and we say that any regression with a single local maximum is an inverted-U, even if it exhibits a more complex shape with many local minima. The shape could be an “M” if two dosages select for resistance or an “L” if low dosages select most for resistance, we now investigate which of these occur in practice.

## Results

The following idea is used throughout to quantify phenotypic rates of adaptation. For any microbial phenotype, *f*, be it population density, growth rate, protein expression levels, or something else entirely, suppose *f* depends on time, *t*, and erythromycin dose, *E*. Changes in *f* correspond to phenotypic changes in antibiotic resistance and numerical derivatives (*a.k.a.* differences) of *f* can be used to quantify rates of resistance adaptation (Hegreness et al. 2008). So, $\partial f/\partial t=(f_{t+1}-f_{t})/\Delta t$ is the change of *f* with respect to time. Applying this idea to data, we can seek the dosage, *E*, for which the rate of change of *f* is greatest and we call these dosing “hotspots” throughout.

### Prediction: Greater Antibiotic Sensitivity Supports Fastest Adaptation at Lower Dosages

Before presenting any data, we first turn to two simple theoretical models to show that whereas inverted-Us likely depend on many parameters, they should be independent of a bacterium’s MIC (Day and Read 2016). To see this, consider the following exactly solvable model of an exponentially growing population:

(1a) $$\frac{d}{dt}S=(g(A)-d)S-\mu S,$$

(1b) $$\frac{d}{dt}R=(\ell g(\lambda A)-d)R+\mu S.$$

Here, *μ* is mutation rate from a drug susceptible ancestral strain (*S*) to a resistant mutant (*R*). We assume an initially clonal population so S(0)=1 and R(0)=0, *g* is a dose-dependent *per capita* growth rate, *d* is a natural death rate and *A* is antibiotic dosage. The MIC of *S* occurs at the dose, *A*, where *g*(*A*) = *d* and the MIC of *R* (*a.k.a.* the mutant prevention concentration [MPC]) occurs where ℓg(λA)=d. The scaling parameter *λ* (between 0 and 1) is a simple device to reduce the drug concentration that *R* experiences relative to *S* but this reduction comes at a fitness cost controlled by ℓ<1 (ℓ multiplies the growth rate of *R*, reducing it).

Define $\theta_{1}:=g(A)-d-\mu ,\theta_{2}:=\ell g(\lambda A)-d$, (1) has an exact solution:

$$S(t)=\text{exp}(\theta_{1}t),\;\;R(t)=\frac{\mu }{\theta_{1}-\theta_{2}}(\text{exp}(\theta_{1}t)-\text{exp}(\theta_{2}t))$$

wherein the frequency of *S* in the population, *ρ*, is independent of *S*’s MIC because $\theta_{1}-\theta_{2}$ is independent of *d*:

$$\rho :=\frac{S}{S+R}=\frac{1}{1+\mu (1-\text{exp}((\theta_{2}-\theta_{1})t))/(\theta_{1}-\theta_{2})}.$$

Now, if $\theta_{2}>\theta_{1}$ then ρ(t)→0 as t→∞ and the resistant mutant approaches fixation. If $\theta_{1}>\theta_{2}$ then mutation-selection equilibrium is reached with $\rho (t)\to 1/(1+\mu /(\theta_{1}-\theta_{2}))$ as t→∞ and the rate of convergence is determined by the exponential in the definition of *ρ*, so we define the selection coefficient (Day et al. 2015)

$$s(A):=\theta_{2}-\theta_{1}=\ell g(\lambda A)-g(A)+\mu .$$

Doses where the maximum rate of adaptation (ROA) are to be sought in data correspond here to the dose where *s*(*A*) is maximal and we call both “hotspot” dosages. By elementary calculus, the maximum of *s*(*A*) occurs either at *A *=* *0, in the limit A→∞ or else where (using a dash ′ to denote a derivative).

(2) $$\frac{ds}{dA}=\lambda \ell g\prime (\lambda A)-g\prime (A)=0$$

and $d^{2}s/dA^{2}=\lambda^{2}\ell g\prime \prime (\lambda A)-g\prime \prime (A)<0$.

**Fig. 1.**
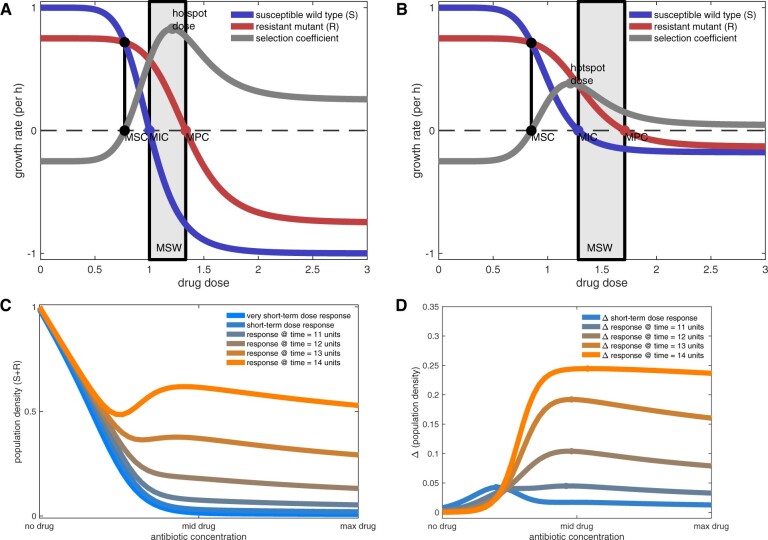
Theoretical inverted-U geometries determined from equations (1) and (3). In equation (1), the MSC (mutant selection concentration) is the smallest dosage where the selection coefficient (*s*(*A*)) is positive so selection for resistance occurs where the gray line is positive. MIC of the ancestral strain, *S*, is the dosage, *A*, where *g*(*A*) = *d* (*g* is the blue curve). MPC (mutant prevention concentration) is the MIC of the mutant strain, *R*. Mutant selection window (MSW)—the range of dosages between MIC and MPC—is where MSW theory predicts selection for resistance occurs. The inverted-U geometry of the selection coefficient can be seen in gray: (*A*) this exemplar has the peak of the inverted-U inside the MSW, (*B*) this example does not. Both (*A*) and (*B)* use the Hill function $g(A)=1/(A^{7}+1)$ but other model parameters differ. (*C)* The dose–response of total population size (*S *+* R*) versus antibiotic dose for equation (3) is monotone for short treatments but nonmonotone for longer ones. (*D*) Inverted-Us obtained by taking the time difference of dose–responses in (*C*) change with the duration of treatment: the dose of most rapid adaptation (the dots) increase with duration.

Hill functions provide standard models for *g* but an exponential decline (Smith and Waltman 1995), g(A)=exp(−pA), controlled by parameter *p* yields a simple and instructive calculation. Solving equation (2) we find,

$$A=A_{\text{hot}}=\frac{1}{p(1-\lambda )}\ln{\left(\ell \lambda \right)}^{-1}$$

which is positive because 0<ℓ,λ<1. $A_{\text{hot}}$ could lie above or below *S*’s MIC because the latter is the solution, for *A*, of *g*(*A*) = *d* which is independent of both ℓ and *λ*. The inverted-U form of *s*(*A*) is illustrated in figure 1*A* and *B*.

Now, if one could use genetic manipulation in practice to synthetically increase sensitivity to the antibiotic, this could increase *p* which would decrease $A_{\text{hot}}$, or, to put it differently, increasing antibiotic efficacy reduces the hotspot dose in this simple model. The hotspot could, therefore, occur close to zero for sufficiently severe genetic perturbations which, for *E. coli* and erythromycin, could entail the removal of *rrl* operons, manipulation of the *mar* regulatory network or loss of function of the *acrAB* operon; we investigate the latter below. Moreover, the dosing hotspot clearly depends on several biological parameters by equation (2) and because different mutations would associate with different modeled parameter values, this logic predicts different resistance mechanisms could have different hotspots.

The unchecked exponential growth of equation (1) is not necessarily realistic so a second model predicts how inverted-Us might appear in empirical population density data:

(3a) $$\frac{d}{dt}S=S\cdot (1-A-(S+R))-\mu S,$$

(3b) $$\frac{d}{dt}R=R\cdot (1-(S+R))+\mu S.$$

As in equation (1a and 1b), *S* is susceptible and *R* is resistant to the antibiotic, *A*, where unit carrying capacity is now assumed. We impose no costs of resistance but one could be included. Now, dose–responses of equation (3a and b) where population density, S(t)+R(t), is plotted against antibiotic, *A*, are not monotone (fig. 1*C*) because those densities increase most rapidly at intermediate dosages. We therefore observe complex inverted-U-like shapes that change through time when we seek the most rapid changes in population density by taking the time difference of the latter (fig. 1*D*). An analytic selection coefficient is not available for equation (3a and b) but these nonmonotone dose–responses are an important feature of *E. coli* in vitro, as we now show.

### Antibiotic Treatments of *E. coli*

We sought inverted-Us in population density data by treating four strains of *E. coli* K12 (AG100, AG100A, TB108, and eTB108) in minimal M9 media supplemented with erythromycin at concentrations 0, 5, 10, …., 50 µg/ml once daily for 7 days (*a.k.a.* seasons, eight replicates per dose). With each round of treatment *E. coli* may experience a lag phase and a period of exponential growth followed by the stresses of resource depletion and stationary phase, or very little growth at all, each of which is mediated by antibiotic (see Materials and Methods, supplementary figs. 16–18, Supplementary Material online). AG100A is a knockout strain derived from *E. coli* AG100 that lacks functional AcrA, *E. coli* MG1655 is the parent of TB108 which has GFP fused to AcrB and eTB108 is derived from TB108 by adapting it to sublethal dosages of erythromycin (see Materials and Methods). Accordingly, all four differ in their dose–responses to erythromycin: AG100A is most sensitive, then TB108, whereas AG100 and eTB108 have similar responses (supplementary fig. 1, Supplementary Material online).

We measured population densities continually during treatment, we also measured efflux protein (AcrB) expression continually for TB108 and eTB108, as explained below. We then quantified evolved dose–responses for the four strains and sought resistance mechanisms by destructively sequencing 3, AG100 metapopulations sampled from three replicate lineages every other day (days 1, 3, and 5). Moreover, spectrophotometry was used to estimate both mean AcrB-GFP and optical density (OD at 600 nm) levels which, because the latter is a unit of biomass that correlates with cell counts (adjusted $R^{2}>0.95$, supplementary fig. 25, Supplementary Material online), dividing AcrB-GFP by OD data provides approximate data on the mean expression of AcrB per cell.

#### Population Density Adaptation Correlates With Changes in Efflux

**Fig. 2.**
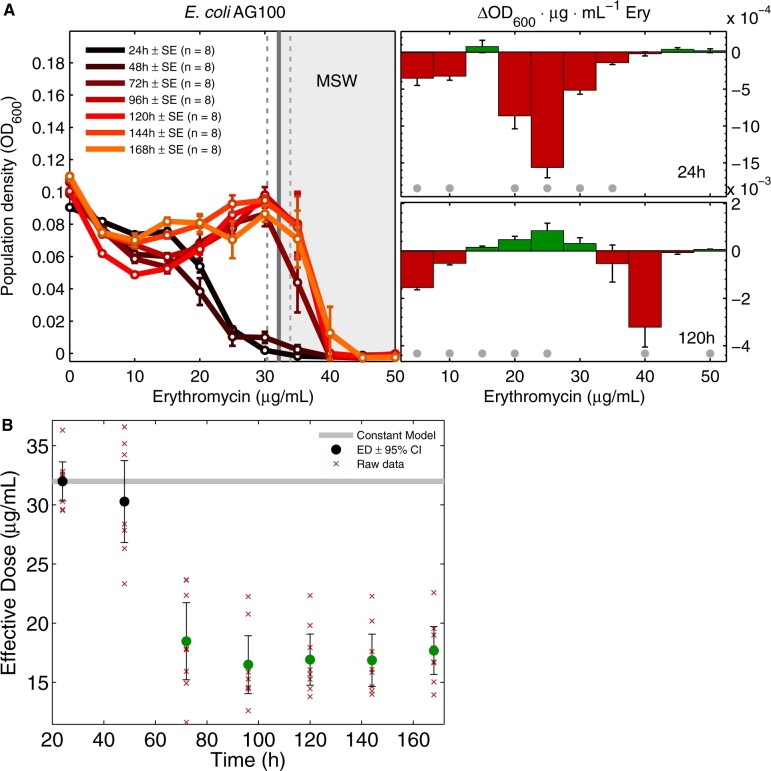
Dynamic dose–response data from erythromycin treatments of AG100 are not monotone decreasing. (*A*) Changing *E. coli* AG100 dose-response profiles are shown for each 24 h season: AG100 lineages are propagated separately at fixed dosages and population size data each day are plotted as if they were dose–responses. Note the most rapid increases near the MIC (the vertical line near 32.1 µg/ml ± 95% CI [dotted vertical lines]) and the hypothetical MSW above the MIC (gray area). Note the lack of monotonicity whereby increments in drug concentration can increase (green bar) or decrease (red bar) bacterial density, as shown in the right subplot after 24 and 120 h of treatment (mean ± SE, *n *=* *8; significant changes (*P *<* *0.05) with respect to the hypothesis of no change (two-sided *t*-test) are gray dots). (*B*) The reduction in EAD (see Materials and Methods) for AG100. The null hypothesis that erythromycin sensitivity does not change through time is marked by the gray line, green dots show this null can be rejected beyond 72 h (two-sided *t*-test, *P *<* *0.05).

The continuous measurement of optical densities (OD, taken at 600 nm, Materials and Methods) as a proxy of AG100 populations’ biomass initially formed a dose–response that, as expected, declined with increasing drug concentration (fig. 2*A*, 24h data), putting the MIC (IC_99_) at 32.1 ± 1.8 µg/ml (mean ± 95% C.I., *n *=* *8; supplementary fig. 1, Supplementary Material online). By season 3, AG100 was detected at dosages where it previously had not been (fig. 2*A*, 72h) and all populations exhibited increasing densities, indicating reduced antibiotic efficacy (fig. 2B).

**Fig. 3.**
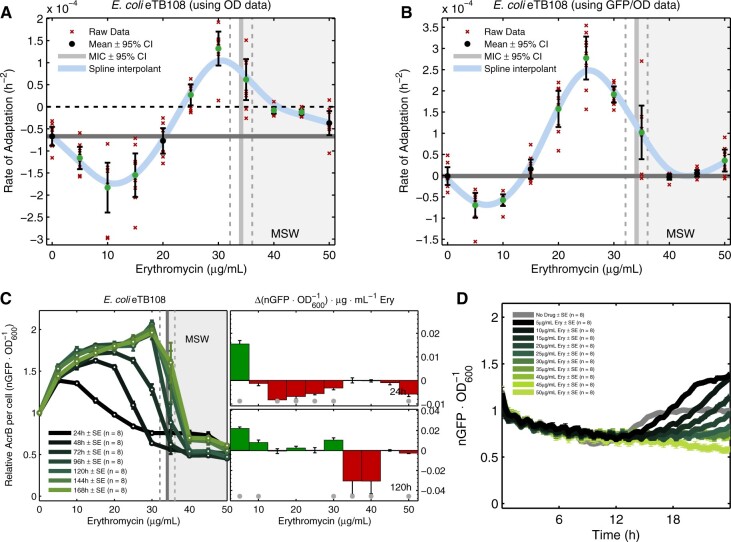
Phenotypic rates of adaptation are inverted-U-like. (*A*) An interpolant of the mean ROA of eTB108 population density (OD, in cyan) peaks near the MIC (supplementary fig. 14, Supplementary Material online, is analogous for AG100). (*B*) Rates of adaptation of population mean AcrB per cell (i.e., AcrB-GFP per OD) clearly correlate with rates of population density adaptation in (*A*) ($R^{2}\approx 0.84$, see supplementary fig. 2, Supplementary Material online). (*A*) and (*B*) show spline interpolants of the data mean (cyan), dashed horizontal lines indicate zero adaptation, thick horizontal lines indicate adaptation rate in the absence of erythromycin and significant changes with respect to that are shown by green dots (two-sided *t*-tests , mean ± SE, *n *=* *8). (*C*) Green lines are treatment-by-treatment experimental dose-response profiles of mean AcrB-GFP per cell (i.e., $\mathrm{nGFP}\cdot OD^{-1}$). The changes drug concentration increments have on AcrB-GFP per OD are shown in the right subplots at 24 h and 120 h (mean ± SE, *n *=* *8): increases are green bars and decreases are red bars. Significant changes in mean AcrB-GFP per OD (two-sided *t*-test) are denoted by gray dots: note the stepwise change at lower dosages from predominantly negative (black) to positive (green) correlations between erythromycin and AcrB per OD. (*D*) Mean AcrB-GFP per OD in the first 24 h season at all erythromycin concentrations used (mean ± SE, *n *=* *8; the oscillations are an electro-mechanical phenomenon, see supplementary fig. 22, Supplementary Material online).

To quantify the rate of adaptation (ROA), we applied a prior rate of change measure (Hegreness et al. 2008) to population densities (see Materials and Methods): the ROA of AG100 to erythromycin has a nonlinear, nonmonotone dependence on dose that exhibits qualitative consistency with equation (3a and b) (c.f. figs. 3*A* and 1*D* [blue line]). Moreover, populations adapted fastest at near-MIC dosages (30 and 35 µg/ml, fig. 3*A*) and the latter figure exhibits an inverted-U. eTB108 behaves analogously (fig. 3) with a high correlation between ROA in population density (OD, fig. 3*A*) and ROA of population mean AcrB-GFP per OD (fig. 3*B*; Deming regression $R^{2}\approx 0.84,P\ll 0.05$, supplementary fig. 2, Supplementary Material online). Thus, increases in AcrB expression correlate with population density increases for eTB108.

In order to quantify phenotypic increases in resistance, one could sample populations each day and determine their respective changes in erythromycin MIC. As this requires a dose-response assay for each replicate of each lineage each day, we reduced the overhead by introducing a measure, the effective antibiotic dose (EAD), which can be determined directly from population density data as it is collected. The EAD (fig. 2*B*) is the dose that if it had been applied to the ancestral strain, it would have resulted in the same population density as the drug-adapted population (see Materials and Methods). For example, if a drug-treated strain grows to the same density as the untreated ancestral strain, then the EAD is zero, but if a treated strain grows to the same density as that achieved by the ancestral strain when cultured at dose *x*, then the EAD *is x*. The EAD can, therefore, be determined from the dose-response of the ancestral strain and just one culture of a treated strain (see Materials and Methods). Now, for the fastest adapting AG100 populations, the EAD reduces by approximately 50% within 72 h (fig. 2*B*).

#### Dynamic, Wave-Like Correlations between Antibiotic Dose and AcrB Expression

We expected the *acrAB* operon would mediate resistance but because erythromycin binds the ribosomal 50S subunit and inhibits protein synthesis (Sparling and Blackman 1973), we first reasoned this inhibition might create a *negative* correlation between dose and AcrB per cell. However, a second expectation was also plausible whereby AcrB per cell could increase with dosage, mediated by the *mar* resistance regulon (Alekshun and Levy 1999). In practice we observe much more nuanced behavior than either of these possibilities whereby dose-dependent, “wave-like” dynamics appeared in the changing correlations between erythromycin and AcrB expression.

To elucidate this relationship, spectrophotometry data approximating mean AcrB per cell (AcrB-GFP per OD) increase at 24 h as drug passes from 0 to 10 µg/ml (fig. 3*C*) but they then decrease as drug is further increased, up to and beyond the MIC. This remains true in late phase bacterial growth (12–18 h, fig. 3*D*). However, this pre24h, predominantly negative efflux pump-drug correlation is replaced in a step-wise manner by an increasingly positive correlation as treatment proceeds which creates a wave-like front in changing AcrB-GFP per OD expression profiles (fig. 3*C* and supplementary fig. 6, Supplementary Material online).

#### acrAB Amplification Correlates With Loss of Drug Efficacy and Increased AcrB Expression

*acrAB* helps bacteria negotiate different phases of growth in vitro, even without antibiotics (Zhang et al. 2011) and, accordingly, AcrB-GFP expression dynamics are qualitatively similar here in the absence and presence of erythromycin (fig. 3*D*). When treated with erythromycin, AcrB expression tends to increase with each treatment round (supplementary fig. 7, Supplementary Material online) although AcrB-GFP per OD first declines each day just after treatment begins (in lag phase) only to increase later through logistic-like dynamics whether erythromycin is present or not (fig. 3D and supplementary fig. 7, Supplementary Material online).

**Fig. 4.**
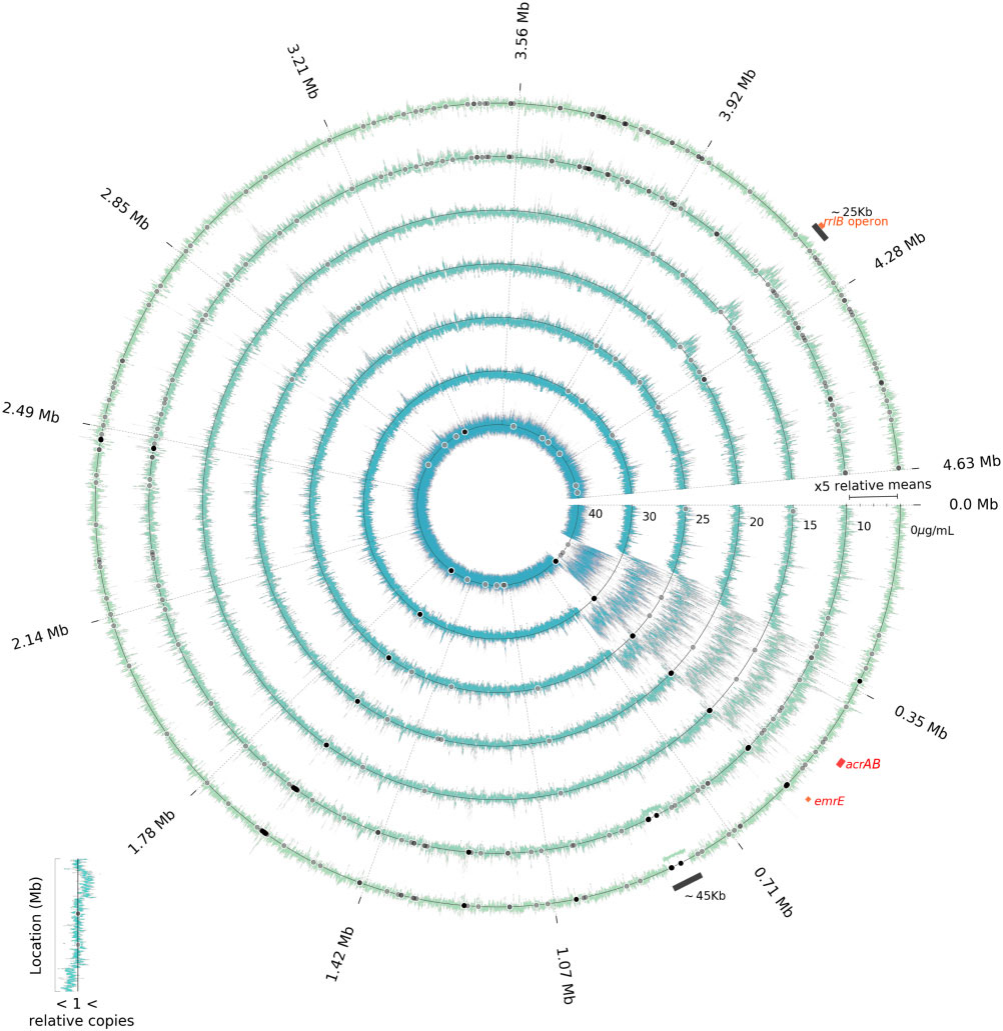
Normalized Illumina coverage of AG100 at different erythromycin concentrations following 5 days’ treatment (one ring per dose). Genomic copy number changes were estimated from normalized coverage data for metapopulations treated at the erythromycin concentrations shown in µg/ml. The outer ring is for populations without erythromycin, subsequent inner rings use increasing concentrations of erythromycin (10–40 µg/ml; for replicates see supplementary figs. 11 and 12, Supplementary Material online). Coverage in excess of the mean for a gene at the start of treatment (“relative copies” > 1) indicates gene amplification, values below the mean indicate gene deletions. Novel SNPs above 5% frequency are black dots, indicating parallelism or divergence between treatments. The amplified operon *acrAB* is highlighted in red and the amplification of a ∼25-kb region between 4,164 and 4,189 kb is highlighted as a black bar (supplementary section 5, Supplementary Material online, describes this region). A deleted 45-kb putative prophage near 0.8 Mb is also highlighted as a black bar.

**Fig. 5.**
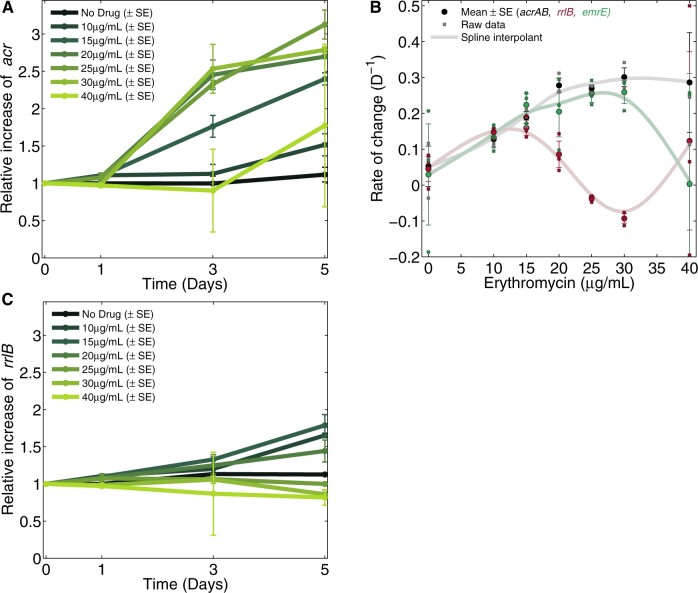
Inverted-U geometries in which *acrAB, emrE*, and *rrlB* amplification rates are maximal at different dosages. (*A*) Increases in relative coverage data are indicative of increased abundance of the *acrAB* operon per genome through time (mean ± SE, *n *=* *3, unity means no change) which occurs at nonzero erythromycin concentrations (color coded doses, see legend). (*B*) Shown are rates of increase in *acrAB, emrE*, and *rrlB* copies per genome versus erythromycin dose: these rates are maximized at 30 µg/ml for *acrAB* and *emrE* and at 10–15 µg/ml for *rrlB*. (*C*) The analogous plot to (*A)* for *rrlB* indicates positive selection for *rrlB* duplications. (*A* and *C* use units of mean relative coverage per genome, [*B*] shows estimates of daily changes in the latter.).

**Fig. 6.**
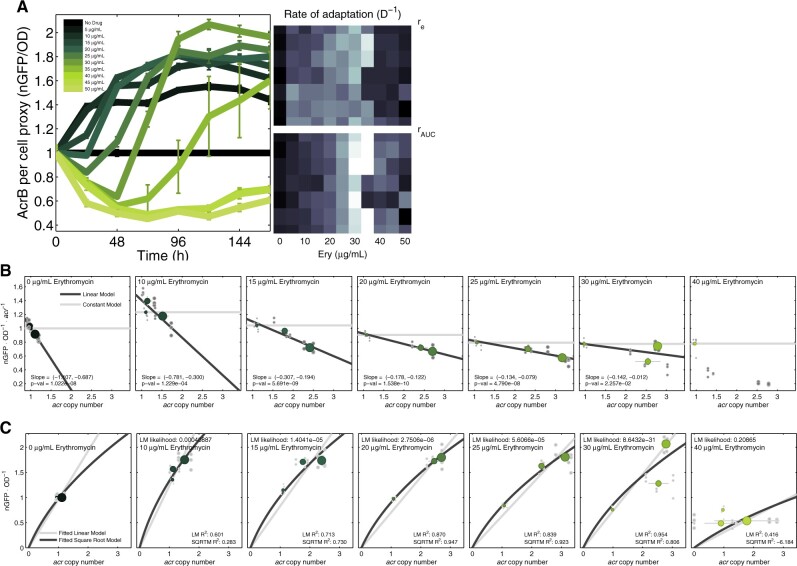
Increases in *acrAB* copies per genome correlate with increases in AcrB expression. (*A*) Temporal dynamics of population mean AcrB-GFP per OD (nGFP/OD—used as a proxy for AcrB per cell) are logistic-like at different erythromycin concentrations (color coded, mean ± SE, *n *=* *8). Rates of increase in AcrB-GFP per OD per day are shown in the rightmost grayscale as heatmaps using two metrics (see Materials and Methods): both are maximized near MIC concentrations (white blocks are fast adaptation, black are slow, eight replicates). (*B***)** Linear regressions of population mean AcrB-GFP per OD per *acrAB* operon (*y*-axis) decrease with *acrAB* operon copy number (“Slope” in legends indicate 95% CI of the regression gradient). Horizontal gray lines indicate the mean AcrB-GFP per OD per operon at the start of treatment (close to unity in all cases, as expected), raw data are gray dots, means and SE (*n *=* *3) for each day are green dots (indicating 24, 72, and 120 h whereby larger dot sizes are later times). (*C***)** AcrB-GFP per OD (*y*-axis) are positively correlated (linear/square root regressions shown) with *acrAB* copies per genome (*x*-axis) at different erythromycin dosages, from no drug (left-most where no correlation is reported due to an absence of *acrAB* duplications) to super-MIC (rightmost).

Changes in AcrB expression are likely to have a genomic basis here because increases in *acrAB* per genome should increase AcrB expression. To quantify this, we used whole genome sequence data to correlate chromosomal copies of *acrAB* with phenotypic AcrB-GFP data, we also compared rates of adaptation of population density with *acrAB* copy number changes inferred this way (see Materials and Methods). These comparisons show 5 days’ erythromycin exposure selects for an amplified 302-kb chromosomal region (Laehnemann et al. 2014) (274–576 kb) containing *acrAB* (480–485 kb) whereby just one copy of this region is observed at all times in the absence of antibiotic (fig. 4 and supplementary figs. 8–10, Supplementary Material online). When drug was present, *acrAB* copy number increased at rates that depended on dosage (fig. 5*A* and *B*) where the highest rate occurred at around 30 µg/ml (fig. 5*B*). These *acrAB* amplifications are correlated with increases in AcrB-GFP expression with significantly positive, nonlinear correlations (AcrB per OD in fig. 6*A*, AcrB per OD per operon in fig. 6*B*, AcrB per OD versus operon copy number in fig. 6*C*).

By comparison, the amplification of a 25-kb genomic region containing the ribosomal RNA operon *rrlB* (a drug target) occurs at a lower rate (fig. 5*C*) than *acrAB*. The efflux operon *acrEF* is implicated in erythromycin resistance (Sulavik et al. 2001) and shares common substrates with *acrAB* but it was not amplified in any treatments (supplementary fig. 15, Supplementary Material online).

#### Regulatory Changes That Increase AcrB per acrAB Operon

As erythromycin inhibits translation, we reasoned the number of efflux pumps per cell per *acrAB* operon could decline with increasing dose and data concur (fig. 6*B*). We then reasoned both *efflux pumps per cell* and *pumps per cell per operon* could increase during treatment due to regulatory adaptation. However, no mutations were observed in the *acrAB* regulators (White et al. 1997; Alekshun and Levy 1999) AcrR and MarR, but we did see the following evidence of regulatory change.

Little daily variability in *acrAB* per chromosome and AcrB-GFP per OD was observed in the absence of erythromycin, both maintained ancestral levels each day (figs. 5*A* and 6*A*). However, they varied during treatment: at 5–10 µg/ml erythromycin, mean AcrB-GFP per OD increased and reached an equilibrium at approximately 50% above ancestral levels (fig. 6*A*). Mean AcrB-GFP per OD increased further at higher drug concentrations, achieving twice ancestral levels (fig. 6*A*). The rate of increase of AcrB-GFP per OD depended on antibiotic and it reached an equilibrium level fastest at dosages close to the MIC (30 µg/ml in fig. 6*A*).

Now, a doubling of AcrB-GFP per OD occurred in populations where *acrAB* per chromosome had tripled (fig. 6*C*). This could result from the amplified repression of *acrAB* by AcrR, or from the lon protease (that degrades MarA), both of which are coamplified with *acrAB*, causing fewer pumps per cell to be produced per additional operon; data are consistent with this in indicating negative correlations (fig. 6*B*). However continued treatment mitigates these negative correlations in the sense that regressions showing pumps expressed per *acrAB* operon (fig. 6*B*) have negative slopes that increase through time towards zero at higher antibiotic doses. Despite this phenotypic evidence of regulatory change, mutations found in *acrAB* regulators in other studies of erythroymycin (Laehnemann et al. 2014) were not observed.

However, regulatory change could occur if an amplified gene has a regulator that is not coamplified. This is relevant here because *acrAB* is repressed by AcrS (also EnvR [Hirakawa et al. 2008]) and, importantly, *envR* coverage data per genome remains constant throughout treatment (supplementary fig. 27, Supplementary Material online). This could increase AcrB per *acrAB* operon by virtue of seeing a reduction in the repressors per *acrAB* operon when the latter is amplified, causing EnvR to compete for additional AcrA sites (Hirakawa et al. 2008). This effect should also have a dose-dependence whereby greater dosages that select for more *acrAB* copies also have more AcrB-GFP per *acrAB* operon and this is consistent with data (fig. 6*B*).

Amplification of the lon protease (at 456–460 kb) inhibits *marA* which could reduce, not increase, expression of *acrAB* (Nicoloff and Andersson 2013). However, this does point to other potential mechanisms of regulatory adaptation because this increases MarA turnover in the mar network whose proteins, MarA, SoxS, and Rob, bind with different affinities to around 10,000 “marbox” sites on the *E. coli* chromosome. Not all of these are regulators of gene expression but many are (Martin et al. 1999; Sharma et al. 2017) and an increase in *acrAB* per genome by one copy increases the length of the chromosome by 7%, creating approximately 2,000 new marboxes in the most rapidly adapting treatments (fig. 4*A*) that could, in theory (Mileyko et al. 2008), mediate *acrAB* expression.

### Other Genomic Amplifications

Coverage data at 24, 72, and 120 h reveal other amplifications selected during treatment. Amplifications of the *dlp12* prophage and efflux pump, *emrE* (Ovchinnikov et al. 2018), exhibit an inverted-U in a manner analogous to *acrAB* when a selection measure is regressed against antibiotic dose (fig. 5*B*). Operon *rrlB* experiences antibiotic-dependent amplification with an inverted-U (fig. 5*B* and *C*), but the hotspot for the *rrl* operons occurs near the subMIC dose of 15 µg/ml erythromycin.

**Fig. 7.**
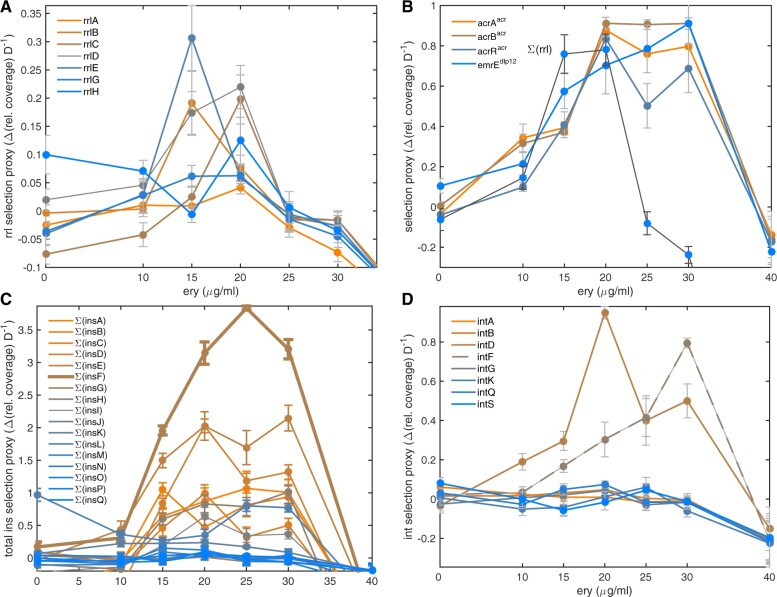
Genes whose “selection proxy” for amplifications have inverted-U geometries. (*A*) Rates of amplification of the *rrl* operons (change in relative coverage of Illumina data, “selection proxy” defined in Materials and Methods) peaks at around 15 µg/ml, below the MIC of 30 µg/ml. Assuming *rrl* amplifications do not occur at zero erythromycin dose, these data define a baseline in between-operon variation that only four *rrl* operons exceed significantly at 15 and 20 µg/ml. (*B*) The selection proxy is shown for *acrAB* and *emrE* genes (where selection proxy data from (*A*) has been summed for all *rrl* operons to provide a visual comparison). (*C*) Selection proxies for insertion sequences where data have been summed across all copies of each sequence in the ancestral genome. (*D*) The integron *intD* has an inverted-U profile in its selection proxy, as does *intF* (dashed line) but the latter is situated close to *acrAB* in the genome and it has a selection profile analogous to that operon (see *B*).

Selection for *rrl* amplifications differs from *acrAB* in several respects. First, *acrAB* resides within a large contiguous region encompassing 7% of the chromosome (of size 302 kb) flanked by, and rich in, IS elements and phage and its amplifications occur at all dosages. By contrast, *rrl* amplifications occur in a narrower dose range of 15–20 µg/ml erythromycin (fig. 7*A*) and *rrlB* resides within a 25-kb region whose amplifications increase the availability of transcription and translation machinery (supplementary section 5, Supplementary Material online; fig. 4) that may help overcome the inhibitory properties of erythromycin. Evidence of selection for *rrl* amplifications is clearest for those operons expressed most in lab media (Maeda et al. 2015) (namely *rrlE*) with weaker evidence for duplications of the *rrl* operon expressed least (Maeda et al. 2015) which does not exhibit an inverted-U (*rrlA*; fig. 7*A*).

The molecular basis for the amplification of the 25-kb region carrying *rrlB* is not well understood. Regions between *rrl* operons can be hotspots for gene amplification (Reams and Roth 2015) and *rrl* operons, as highly expressed regions, can stall DNA replication. Stress can be also a trigger for gene amplifications because starvation-induced stalling of DNA replication (Slack et al. 2006) can lead to the emergence of 3’-single stranded DNA ends. These are prone to template-switching, generating direct repeats of 7–32 kb bordered by 4–15 base pairs of G-rich microhomology (Slack et al. 2006). We found some evidence of this whereby one sequence, GTGGCTGG, lies at the edges of the 25 kb amplified segment although this microhomology is also relatively frequent throughout the chromosome. More work is therefore needed to establish the molecular mechanism that amplifies *rrlB*.

**Fig. 8.**
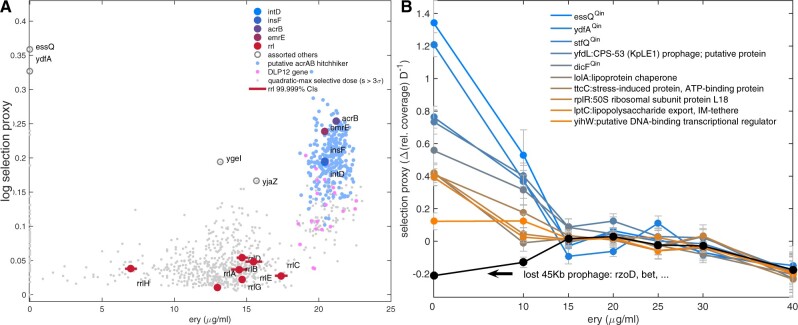
Amplifications of different genes are maximized at different erythromycin dosages. (*A*) Let *s*(*E*) denote the selection proxy for the amplification of a gene at erythromycin dose *E* (see Materials and Methods). *E* is shown on the *x*-axis and the log of the estimated maximum of the selection proxy is shown on the *y*-axis for every gene [one dot per gene 3 SD above 0 as predicted from a quadratic regression of *s*(*E*), Materials and Methods[: $\text{log}\;\left(\max_{E=0\ldots 40\mu g/\text{ml}}s(E)\right)$. Two clusters are apparent: one (blue and pink) shows the selection proxy is greatest near 20–25 µg/ml for genes coamplified with *acrAB* and *emrE*. The *dlp12* prophage is in this cluster, as are insertion sequences (*ins*) and integrons (*int*). This cluster is below, but near, the erythromycin MIC whereas the cluster of *rrl* operons (dark red dots) exhibits maximal selection at lower dosages. Selection for assorted other genes is observed at mid and low dosages, including the prophage Qin containing *essQ* at zero dosage. (*B*) The genes shown here for which *s*(*E*) exhibits the largest decreases with dose may be indicative of media adaptation not resistance, like Qin genes. The deletion of a 45-kb region encoding phage genes *rzoD* and *bet* sweeps close to fixation within 5 days (black line) but only in the absence of erythromycin.

Figure 7 summarizes selection data (“selection proxy” defined in Materials and Methods) for the amplification of *rrl* operons, *acrAB* genes, *emrE*, *ins* insertion sequences and *int* integrons. Where there are multiple copies of any of these in AG100, selection data are summed across all copies for visualization purposes, denoted in figure 7 by a sigma, Σ. Of these, figure 7 highlights genes whose copy number changes have inverted-Us, where all have dosages of fastest increase ranging from 15 to 30 µg/ml. Conversely, figure 7 highlights genes that do not exhibit an inverted-U. To investigate how selection for gene amplifications varies across the genome, we estimated the dosage at which the possible amplification rate of every *E. coli* gene was maximal (see Materials and Methods). These data have several gene clusters (fig. 8*A*): one containing the *rrl* operons (with hotspot around 15 µg/ml), one containing *acrAB* (hotspot close to 20 µg/ml) and one containing the *dlp12* prophage (hotspot close to 20 µg/ml). Amplifications of the Qin prophage, however, do not have an inverted-U and instead see their selection data decline with dose (fig. 8*B*). We therefore reason Qin may be implicated in adaptation to growth media.

#### Single Nucleotide Polymorphisms

**Fig. 9.**
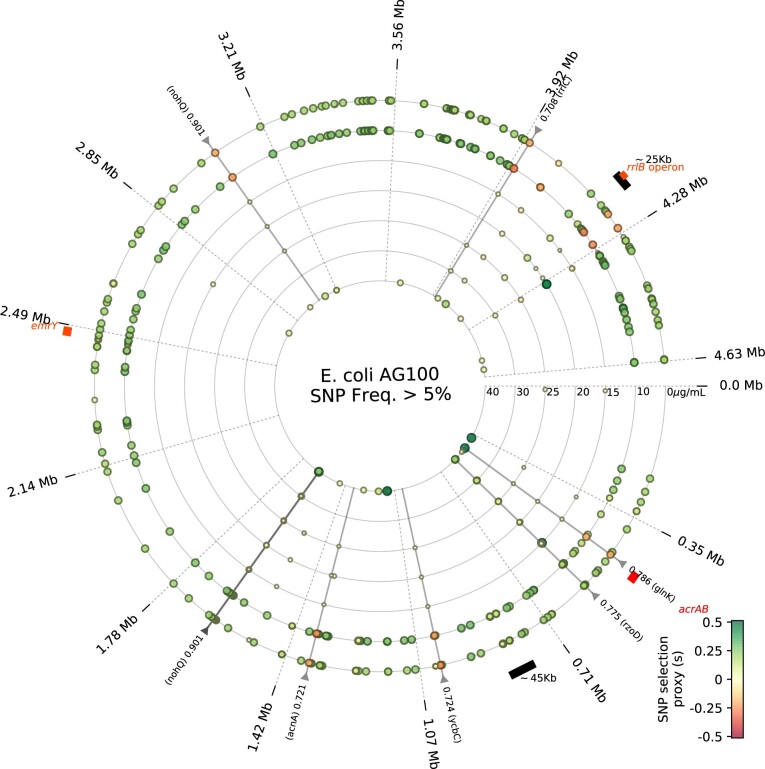
AG100 SNPs at different erythromycin dosages following 5 days’ treatment. Dots are SNPs whose colors indicate a proxy measure of selection, *s*, as shown in the legend colorbar (see Materials and Methods). The outermost ring shows zero erythromycin dose, inner rings have greater dosages, as indicated. Triangles in the perimeter indicate a between-treatment parallelism metric based on day 5 SNP frequencies (see Materials and Methods), parallel evolution (parallelism coefficient above 0.7, Materials and Methods) is highlighted by a black radial line. A 45-kb deletion and a 25-kb amplification are indicated in the perimeter using black bands, the location of *acrAB* is a red band. Divergence between low and high dosage treatments is apparent from differences in the abundance of novel SNPs found at high and low dosages (supplementary fig. 23, Supplementary Material online).

De novo single nucleotide polymorphisms (SNPs) observed significantly above 5% frequency at 120 h indicate parallel, between-dosage adaptation (supplementary section 6, Supplementary Material online, fig. 9). A clinical case of *acrAB* evolution found protein structure SNPs (Blair et al. 2015), but we observed none. SNPs were observed in the ribosomal RNA operon *rrlC* in all treatments that also occurred in the absence of antibiotic, suggesting media adaptation. We found analogous putative media adaptations at all drug concentrations with SNPs in *fbaA* (a glycolytic enzyme), *glnK* (a nitrogen assimilation regulator), *acnA* (an RNA-binding oxidative stress regulator) alongside substantial polymorphisms in phage genes *rzoD* (a lysis lipoprotein), *ycbC* (an envelope biogenesis factor) and *nohQ* (a DNA packaging protein) which exhibit between-treatment parallelism (see Materials and Methods; supplementary figs. 11 and 12, Supplementary Material online, show SNPs from different biological replicates). SNPs above 5% in frequency at 120 h that are unique to each antibiotic treatment (supplementary table 6, Supplementary Material online) indicate changes in amino acid transport and biosynthesis (genes *tauA* and *putP*).

It was not possible to use these data to determine an inverted-U geometry for a resistance SNP as this would need a highly parallel SNP observed in all drug treatments that both increases the strain’s MIC and which does not arise in the absence of the antibiotic. However, we found no genes satisfying these criteria.

#### AG100A and TB108 Do Not Have Inverted-Us on the Same Timescale as AG100

Consistent with the behavior of equations (1) and (3) when resistance mutations are rare, so R(0)=0, or R(0)>0,μ>0 are small and treatments are short, laboratory treatments of the AcrA-deficient strain AG100A did not yield an inverted-U and neither the dose–response monotonicity nor the EAD changed significantly during treatment (supplementary figs. 3 and 5, Supplementary Material online). This is consistent with equation (2) when inhibition increases rapidly with dose, which AG100A does experience (supplementary fig. 1, Supplementary Material online), where most rapid adaptation occurs at the lowest dosages (cf. supplementary fig. 3, Supplementary Material online). We asked whether this absence of the inverted-U could depend on the duration of treatment.

To address this, we noted that strain TB108, with GFP fused to AcrB (Bergmiller et al. 2017), has a lower erythromycin MIC than AG100 (supplementary fig. 1, Supplementary Material online), so the fusion may reduce efflux efficacy and moreover, like AG100A, TB108 did not initially produce an inverted-U when treated (supplementary fig. 4, Supplementary Material online). So, we sought to improve TB108 pump function by treating it with erythromycin at a subMIC dose until its MIC had increased to that of AG100 (see Materials and Methods, supplementary fig. 1, Supplementary Material online). This resulted in a strain (eTB108) which, upon applying the 7-day erythromycin treatment protocol to anew, *did* exhibit an inverted-U with a peak near its MIC (supplementary fig. 13, Supplementary Material online). Thus, the presence, or absence, of the inverted-U can depend on the duration of treatment.

#### An Observation on Cell Size

*E. coli* can filament when treated with erythromycin (Lau and Zgurskaya 2005) but single cell imaging of eTB108 shows, instead, that it decreases by approximately 18% in median size following subMIC erythromycin treatment (20 µg/ml, Materials and Methods; supplementary fig. 20, Supplementary Material online, ANOVA P≈0.0016). Cell size reductions could be the result of erythromycin reducing protein content per cell, more speculatively they could result from selection for decreases in cell volume which would increase the efficiency of efflux pumps.

Single cell image data are consistent with the approximate doubling seen in AcrB-GFP per OD expression data posttreatment (median change ≈184%, ANOVA $P\ll 10^{-10}$, supplementary fig. 21, Supplementary Material online), corroborating expression data obtained from spectrophotometry (20 µg/ml erythromycin data in fig. 6*A* and supplementary fig. 21*B*, Supplementary Material online, are consistent).

## Discussion

Microbial competition assays have demonstrated that resistance can be selected from low dosages upwards (Gullberg et al. 2011; Liu et al. 2011) and our data are consistent with this: we observe selection for some resistance mechanism at all positive dosages assayed. Conversely, if we assume the MSW hypothesis (Baquero and Negri 1997; Tam et al. 2007) extends without modification to amplification mutants, then this would predict selection for resistant subpopulations should be positive above the ancestral MIC dose. However, our data accord with a growing body of environmental, in vitro, in vivo and theoretical evidence which claim the MSW hypothesis is “technically incorrect” (Zhu et al. 2012; Day et al. 2015; Westhoff et al. 2017; Stanton et al. 2020) and, to illustrate this, the MSW-predicted MSWs are marked in figures 2 and 3. Indeed, the hotspot dosage of most rapid selection for *rrl* and *acrAB* in our data occur below the ancestral MIC.

Similarly, we expected little population density adaptation above the ancestral MIC and this was the case but the super-MIC recovery of some populations and some SNPs were observed (supplementary figs. 16–19, supplementary section 6, Supplementary Material online, Table 6). So, how high must dose be so that no population recovery is observed at all? To answer this, we found that dosing erythromycin each day at 2xMIC produced no detectable growth (supplementary fig. 24, Supplementary Material online) at an approximate 10^5^ cells per ml detection limit (Pena-Miller et al. 2014, supplementary fig. 1*b*, Supplementary Material online).

We have shown that whether the inverted-U is seen in phenotypic data, or not, exhibits subtleties because the timescales over which it appears can be contingent on a single gene. When it is absent, phenotypic rates of adaptation could both increase or decrease with drug dose which is also a feature of genomic data: a poorly annotated 45-kb genomic region was deleted whereby the mutants sweep to fixation but only in the absence of erythromycin but this region was maintained in the drug’s presence (black line, fig. 8*B*). The region harbors phage genes *rzoD* and *bet* and BLAST searches reveal high sequence similarity to known phage genes but why erythromycin should select for their deletion is unclear.

Increased *acrAB* expression has occurred during clinical treatments of sepsis (Blair et al. 2015) where a 2-fold increase in AcrB was detected after 1 week of antibiotic therapy of *Salmonella typhimurium*. Our data exhibit similar timescales because a 3-fold amplification of *acrAB* occurs in a week. However, the 256x increase erythromycin MIC and structural SNPs seen in the patient are not observed here, indicating factors such as cross-resistance from multidrug therapy or the immune response may play a role in changing the timescales over which resistance develops in the clinic relative to our in vitro assays. Indeed, an antibiotic gradient in tissues (Fischman et al. 1998) is a feature of clinical treatment that is absent here and which could hasten resistance (Hermsen et al. 2012; Baym et al. 2016).

Competitive release (Wargo et al. 2007) has been invoked to explain nonlinear patterns in drug resistance (Pena-Miller et al. 2014) and it may be relevant to understanding nonmonotone dose–responses (figs. 1*C*, *D*, and 2*A*). The idea is this: the competition a resistant subpopulation experiences with a drug-susceptible subpopulation is a mechanism that suppresses population growth. Removing, or releasing, that competition by applying an antibiotic could cause the population as a whole to grow more than if less drug were used. The dose of most rapid adaptation can, therefore, be viewed as a sweet spot between how the antibiotic selects for resistant cells and how it reduces competition with drug-susceptible cells. This argument is not specific to our study system and, if true, it indicates that nonmonotone dose–responses and inverted-Us should not be peculiar to erythromycin. Indeed, prior *E. coli* data (Reding-Roman et al. 2017, supplementary fig. 13, Supplementary Material online) shows these features in doxycycline treatments too.

Finally, it was *essential* that treated isolates were not cultured in the absence of antibiotic prior to DNA extraction. Had we done so, data on growth rate costs of *acrAB* amplification (Laehnemann et al. 2014) indicate genomic amplifications could have been lost within 24 h. Having avoided that by sequencing treated populations destructively, our data demonstrate how structural and single nucleotide variation can be mediated by an antibiotic to create subMIC dosages of most rapid change, whether the latter is measured in terms of population densities, gene expression levels or rates of genomic amplification.

## Materials and Methods

### Culture Conditions, Strains and the Drug-Treatment Assay

Strains *E. coli* K12 AG100, AG100A (Δ*acrAB*) were gifts from Stuart B. Levy. eTB108 is a descendant of TB108 isolated from a drug-treatment assay described below where TB108 (Bergmiller et al. 2017) (MG1655 *acrB-sfGFP-FRT*) provides AcrB data from different types of GFP (green fluorescent protein) measurements. All strains were cultured in liquid M9 minimal media supplemented with casamino acids for all controls and drug treatments.

M9 was prepared by mixing dilute K_2_HPO_4_ (350 g), KH_2_HPO_4_ (100 g) in 1 L of de-ionized water and dilute trisodium citrate (29.4 g), (NH_4_)_2_SO_4_ (50 g), and MgSO_4_ (10.45 g) in 1 L of de-ionized water, autoclaved and diluted accordingly. Liquid M9 was supplemented with 0.2% (w/v) of glucose and 0.1% casamino acids from a filtered, sterilized 20% stock. When cultured, all strains were incubated at 30°C, shaken linearly in a microtitre plate reader (spectrophotometer) and propagated in 24 h seasons with one drug treatment at the start of each season. Optical culture densities (OD) for all strains were read at 600 nm and GFP fluorescence measurements were taken for TB108 and eTB108 at 493/526 nm (excitation/emission wavelengths) with readings every 20 min.

For antibiotic treatments at different dosages, a linear gradient of erythromycin (Duchefa) ranging from 0 up to a fixed, typically super-MIC, dose across a 96-well microtitre plate was created by supplementing M9 media with different concentrations using prepared stock. Replicate cultures were incubated for 24 h in 150 µL of media at that erythromycin concentration and ODs of these cultures were read to provide erythromycin dose–response data from where the MIC was determined using only 24 h data.

To prolong erythromycin treatments, *E. coli* was sampled from each of the 96 wells and transferred to a fresh microtitre plate to permit another 24 h season of treatment using a 96-pin replicator (Nunc), these transfers were extended for as many days as required. The same erythromycin concentration was maintained for each propagated population by using a consistent layout for the microtitre plate each day, optical density and fluorescence readings were taken continually at 20 min intervals. Table 1 indicates all treatments used, the *E. coli* strains and erythromycin dose ranges.

**Table 1:** Summary of Strains and the Assays in Which They Were Used: AG100 and AG100A Provide OD data, TB108 and eTB108 Provide OD and GFP Data, AG100 Was Destructively Sequenced Using Illumina Technology.

*E. coli* Strain	Assay	Outcome
AG100	7d treatment/0 to 50 µg/ml ery	Observed inverted-U
AG100A	7d treatment/0, 0.167… 2 µg/ml ery	No inverted-U
TB108	7d treatment/0, 5… 50 µg/ml ery	No inverted-U
	5d treatment/10 µg/ml ery	Isolated eTB108
eTB108	3d treatment/20 µg/ml ery	Isolated eeTB108
	7d treatment/0, 5… 50 µg/ml ery	Observed inverted-U
eeTB108	Single-cell fluorescence microscopy	GFP/cell size changes wrt eTB108

Optical density and fluorescence data were blank corrected by fitting the following three growth models to experimental data (raw data is shown in supplementary figs. 16–19, Supplementary Material online). If *B*(*t*) denotes bacterial OD at a given time, *t*, as measured by spectrophotometry, these models are blank-corrected linear growth relevant for lag phase, blank-corrected exponential growth relevant for the exponential phase and blank-corrected logistic growth for resource- or antibiotic-limited growth, respectively:

(4) $$B(t)=B_{0}+r\cdot t,\;\;\;\;B_{0}+ae^{rt}\;\;\text{or}\;\;B_{0}+K/(1+a\cdot e^{-rt}).$$

*B*_0_ is a constant blank parameter inferred from fitting *B* to OD data. Parameter *r* is an estimated growth rate, *K* is a carrying capacity and *a* is a coefficient whereby $B(0)=B_{0}+K/(1+a)$ which establishes that *a* is inversely related to innoculum density, *B*(0). We implemented a data-fitting algorithm in MATLAB that chose the most appropriate model from the corrected Akaike Information Criterion (AICc). We then blank-corrected the data using the corresponding *B*_0_ that resulted from the optimal datafit.

If B(t;E) denotes bacterial densities at time *t* cultured in erythromycin, the minimum inhibitory concentration (MIC) is the concentration, *E*, satisfying B(24h,E)=B(24h,0)/100, that is, the amount of drug required to inhibit 99% of the growth observed in the absence of antibiotic (*a.k.a.* the IC_99_). These values are estimated (with error bars) by fitting a Hill function to dose–response data (supplementary fig. 1, Supplementary Material online).

#### Treating TB108 SubMIC to Recover AcrAB-TolC Function

We found the erythromycin MIC of TB108 to lie between the MICs of AG100 and AG100A (supplementary fig. 1, Supplementary Material online), suggestive of a reduction in function of AcrAB-TolC due to the GFP-AcrB fusion. To attempt to recover the AcrB function, we therefore propagated a culture of TB108 in 10 µg/ml of erythromycin, subculturing it for 5 days, tracking daily changes in MIC (see Table 1). We considered the complex GFP-AcrAB-TolC to be functional when the MIC of the evolved strain, a new strain called eTB108, was restored to a value close to that of AG100. Supplementary figure 1, Supplementary Material online, shows eTB108 and AG100 have similar dose–responses to erythromycin, both with significantly higher MICs than TB108. Supplementary figure 21, Supplementary Material online, uses fluoresence micropscopy image data for single cells sampled from an adapted population to corroborate spectrophotometry data using a different measurement technique. These image data demonstrate that eTB108 doubles its per-cell GFP-AcrB expression level when propagated in erythromycin so we used eTB108 to quantify changes in GFP-AcrB expression using spectrophotometry.

#### Whole Genome Sequencing

DNA was extracted destructively from adapted populations, importantly without any additional culture steps following antibiotic treatment, and was fragmented by sonication using a Biorupter for 30 s on, 90 s off, using low power for 10 min on ice. Libraries were prepared using SPRIworks cartridges for Illumina (Beckman Coulter) and Nextflex indexed adapters, with 300–600 bp size selection, amplified with eight PCR cycles using Kapa HiFi DNA polymerase and purified using GeneRead kit (Qiagen). Concentrations were determined using a Bioanalyser 7500 DNA chip. Libraries were pooled in equimolar amounts, denatured, diluted to 6.5 pMol and clustered on a flowcell using a cBot (Illumina). 100 paired end sequencing with a custom barcode read was completed on a HiSeq 2500 using Truseq SBS v3 reagents (Illumina).

Reads were processed with fastq-mcf (Aronesty 2013) to remove adapters from data and to trim and filter low-quality reads. Cycles with at least 1% of Ns were removed (command-line parameter: -x 0.01). The remaining reads were mapped to the AG100 reference genome using the Burrows-Wheeler aligner BWA (Li and Durbin 2009; Li 2013) with standard parameters. The resulting alignments were processed with Samtools 1.3 (Li 2011), with pair/trio calling enabled (command-line parameter: -T). Subsequently, alignments were sorted, artifacts and duplicates were removed and finally the alignments were indexed. Unaligned reads were stored separately.

Copy number variation was detected by analyzing coverage per base as measured by Bedtools (Quinlan 2014) after normalizing against the mean genome coverage. Mean coverage depths ranged from over 200 for genes not amplified under treatment to over 400–500 for amplified regions (supplementary fig. 26, Supplementary Material online, shows exemplar raw coverage data). Different coverage normalizations were tested for their robustness in statistical analysis (based on the use of median, mean or mode coverage for regions under study) and no qualitative inconsistency was found, although unimportant quantitative differences from different normalizations were, of course, observed.

To ensure robustness of SNP reporting we used VarScan (Koboldt et al. 2012) and Samtools 1.3. SNPs were detected using VarScan with the following parameters: *P*-value threshold of 0.05 for calling variants, minimum read depth of 20 to make a call at a position, base quality not less than 20 at a position to count a read, frequency to call homozygote of at least 0.9 (command-line parameters: –*P*-value 0.05 –min-coverage 20 –min-avg-qual 20 –min-freq-for-hom 0.9). Samtools used multiway pileup skipping alignments with mapQ smaller than 1, filtering tags for read depth of the alleles (total, in reverse and forward strand), and Phred-scaled strand bias *P*-value (command-line parameters: mpileup -q1 -t DP -t SP -t AD -t ADR -t INFO/ADF -t INFO/AD -t INFO/ADR), then bcftools (Li 2011) with the consensus caller option (-cv) and we filtered SNPs with frequency ≥0.05 in Python. Insertion and deletion mutations/structural variants were detected using Pindel with standard parameters.

#### Quantifying Selection and Between-Dosage Parallel Genomic Adaptation

We determined dN/dS data for SNPs in all populations but did not find it helpful for quantifying relationships between dose and ROA (Kryazhimskiy and Plotkin 2008). To quantify selection for SNPs, we fitted the logistic function

(5) $$f(t)=1/(1+p\cdot e^{-st})$$

to longitudinal SNP frequency data. We then report the value of *s* (or log(s)) as a numerical proxy for the strength of selection (in fig. 9 and supplementary table 6, Supplementary Material online, of SNPs).

Between-treatment parallelism for SNPs observed in gene *g* at some fixed timepoint was quantified by first determining the vector $f=(f_{1},f_{2},\ldots ,f_{N})$ of frequencies at which that SNP was observed for all of the *N *=* *7 different dosages assayed. A parallelism coefficient was then defined as the Euclidean distance, P, of ***f*** from the one-parameter family of uniform vectors of the form λ·(1,1,…,1), namely $P(g)=\min_{\lambda >0}\|f-\lambda \cdot (1,1,\ldots ,1){\|}_{2}$. If *G* represents a set of genes of interest, we determined the gene exhibiting greatest between-treatment parallelism, $P^{*}=\max_{g\in G}P(g)$, and we then define a coefficient of parallelism, *p*(*g*), relative to that maximum by $p(g)=P(g)/P^{*}(g)$. A value of p(g)>0.7 was used to highlight parallelism where *G* is restricts to sufficiently common SNPs, namely all SNPs with frequency, *f_j_*, satisfying $f_{j}>0.05$. This thresholding criteria is arbitrary and was merely chosen so fig. 4*B* would legibly indicate SNPs commonly found in different drug treatments.

We computed a proxy measure for selection of genomic amplifications by modeling increases in relative Illumina coverage data for a genomic region of interest for t≥0 using the function

(6) $$F(t)=\frac{p_{1}(1+t)}{1+p_{2}(1+t)}.$$

Here, *p*_1_, *p*_2_ are unknown parameters to be determined from data and *t* is time in days (*D*). We fitted (6) to coverage data normalized relative to a genomic regions’ mean coverage as observed in the ancestral strain (see fig. 5*A* and *C*), we then determined the maximum derivative of the fitted *F*(*t*). This derivative quantifies the change in relative coverage and is called the *selection proxy* in figures 7 and 8. This value, call it *s*, depends on erythromycin dose, so we write *s*(*E*), with *E* denoting erythromycin dose. Now, *s* determined at E=0,5,10,…,40μg/ml provide data to which a quadratic regression was applied to predict the dosage, *E*, at which *s*(*E*) was maximal. We plotted this dose for each gene, restricting to those for which this value of *s* was 3 SD above zero (fig. 8). A logarithmic *y*-axis is used in figure 8*A* to aid visualization.

#### OD${}_{600nm}$ versus Colony Forming Unit (CFU) Tests for eTB108 (supplementary fig. 25, Supplementary Material online)

eTB108 was grown in liquid M9 (see above) for 24 h in 0, 10, and 20 µg/ml erythromycin. Sequential 2-fold dilutions into nutrient-free M9 were performed, followed by optical density readings at 600 nm. To quantify the colony forming units (CFUs), each culture was further diluted by $10^{-4}$ and $10^{-5}$ in triplicate, 25 ml of each dilution was spread onto LB agar plates that were incubated at 30 °C for 24 h and colonies counted manually.

#### NonBiological Oscillations in Spectrophotometry Fluorescence Data

Oscillations in AcrB-GFP expression data were observed (fig. 3*E*) on the timescale of ∼1 h so we sought a mechanism supporting these. We used the discrete Fourier transform to quantify their dominant frequencies using the fluorescence data in figure 3*E* (fft in MATLAB 2020). Two dominant subharmonics were observed with wavelengths of ∼10 h and ∼0.75 h but control cultures not inoculated with bacteria only exhibited the latter (supplementary fig. 22, Supplementary Material online). The long wavelength harmonic corresponds to a slow timescale of up-regulation of *acrAB* in exponential phase whereas the high frequency harmonic, as it was observed in the absence of biological material, must be an electro-mechanical phenomenon due to the microtitre plate reading device.

#### Quantifying Rates of Adaptation

We determined ROA statistics using a method defined elsewhere (Hegreness et al. 2008). The ROA quantifies the rate of change of any dynamically observable phenotype which could entail longitudinal GFP data or population densities estimated from OD. Suppose *r*(*t*) denotes OD at time *t *>* *0, the adaptive time (Hegreness et al. 2008), *t_a_*, satisfies $r(t_{a})=r(0)+\Delta r/2$ where Δr=r(T)−r(0) and *T* is the time at the end of treatment. The ROA is defined to be $\alpha :=(\Delta r/2)/t_{a}$. Figure 3*A* is the result of plotting *α* at different antibiotic concentrations. We also determine ROAs where the timeseries, *r*(*t*), is replaced with GFP per OD data which serves a proxy for population mean AcrB per cell (fig. 3*B* and *C*).

As bacterial cultures with antibiotics are not necessarily well captured by one growth law, we verified the robustness of the ROA of OD by implementing two additional methods. The first was a forward difference approximation to derivatives of *per capita* growth rate, called *r_e_* below, that we applied to the best AIC-model fit, *B*(*t*), determined by fitting (4) to OD data. The second method uses a reciprocal area under the curve (*r*_auc_) measure which has units of $h^{-1}$ to reflect a per capita growth rate. These values are defined for each 24 h season of growth as follows:

(7a) $$r_{e}=\max_{0\leq t\leq 24h}\frac{1}{B(t)}\cdot \frac{B(t+\Delta t)-B(t)}{\Delta t},$$

where Δt=20 min because bacterial densities are read with that frequency, and

(7b) $$r_{\text{auc}}=B(24h)/\int_{0}^{24h}B(t)\cdot dt.$$

ROAs computed using *r_e_* are shown in supplementary figures 13 and 14, Supplementary Material online, uses *r*_auc_ and both exhibit similar behavior (fig. 6*A* shows both, see black and white heatmaps).

The *r_e_* measure could be applied directly to raw data if *B*(*t*) represented an empirical, longitudinal population density data set but applying a mathematical datafit first, as we do, yields more robust statistical estimates by filtering high-frequency noise. This is important because growth rate is a derivative of *per capita* population density and noise would be exacerbated when taking this derivative.

#### The EAD

The EAD (fig. 2B and supplementary fig. 5, Supplementary Material online) quantifies changes in drug efficacy without measuring the MIC of an adapted populations. To do this here would have needed ∼700 dose–response assays if done for all replicates, all dosages and all days. We therefore motivate the EAD with this question: an “ancestral” bacterium is treated with antibiotic for *t* time units at antibiotic dosage $A_{*}$, growing to population density $B_{*}$. How much antibiotic would have been needed to limit growth of the ancestral bacterium to $B_{*}$ units? The answer is the EAD.

In detail, given a dose-response, *B*(*A*), of population density versus antibiotic for the ancestral strain, and given that strain following treatment which grows to density $B^{*}$ (after 24 h, say), this question asks that we find the value $A_{\text{EAD}}$ for which $B(A_{\text{EAD}})=B_{*}$. Now B(·) can be represented mathematically as a decreasing function, often as a Hill function, and the latter have well-defined inverse functions and so we define $A_{\text{EAD}}=B^{-1}(B_{*})$, where $B^{-1}$ is the inverse of *B*. This ensures the larger $B_{*}$ is, the smaller $A_{\text{EAD}}$ becomes.

## Supplementary Material

Supplementary data are available at *Molecular Biology and Evolution* online.

## Supplementary Material

msab025_Supplementary_Data
