# Supplementary material for: The Antibiotic Dosage of Fastest Resistance Evolution: Gene Amplifications Underpinning the Inverted-U
Source: Mol Biol Evol. 2021 Mar 8;38(9):3847–63. doi: 10.1093/molbev/msab025 (PMC8382913; doi:10.1093/molbev/msab025)
Supplement: msab025_Supplementary_Data [file msab025_supplementary_data.pdf]

4 Supplementary Figures

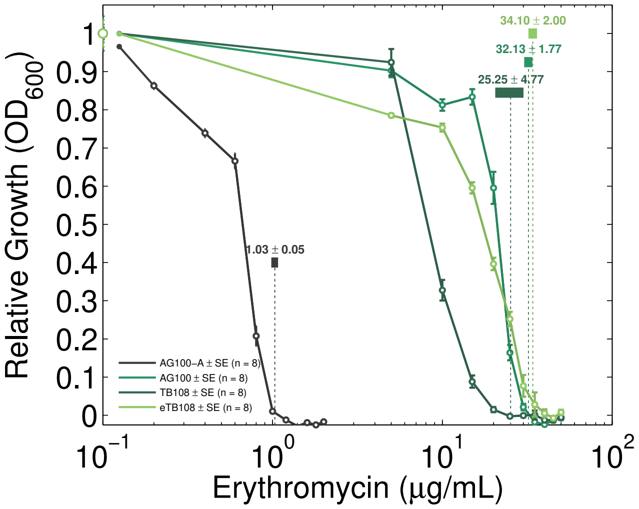

FIGURE S1. Dose-response profiles for all the strains used. 4 erythromycin dose responses for strains *E. coli* AG100, AG100A, TB108, and eTB108 where optical density data has been measured at 600nm (OD_{600}) after 24h of growth. OD_{600} is shown on the y-axis whilst the concentration of erythromycin is represented in a logarithmic scale on the x-axis. The IC_{99} and its 95% confidence intervals ($n = 8$) are indicated as horizontal bars. (IC_x is the dosage at which the population density of a strain is reduced by x%.)

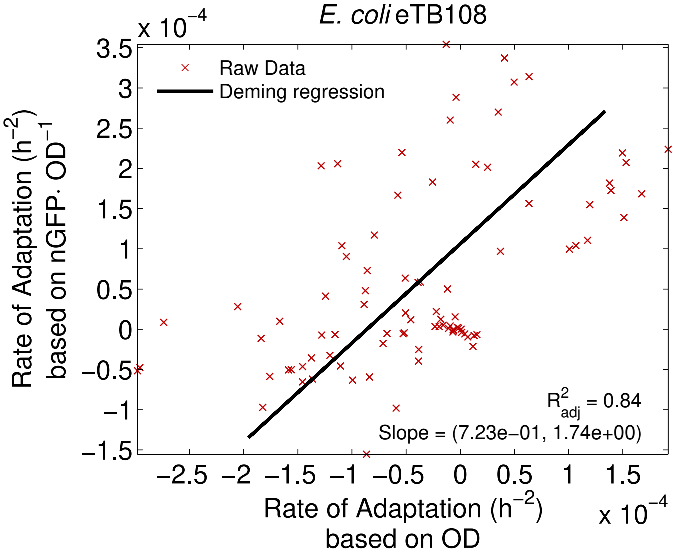

FIGURE S2. The union of the entire adaptation dataset of eTB108 collated at all drug dosages shows the rate of adaptation of population density (based on OD) correlates significantly and positively with the rate of adaptation of mean AcrB-GFP per OD (as measured by spectrophotometry, Deming regression, linear slope parameter 95% CI ≈ (0.72, 1.74)). These data are shown in Figures 3A and B.

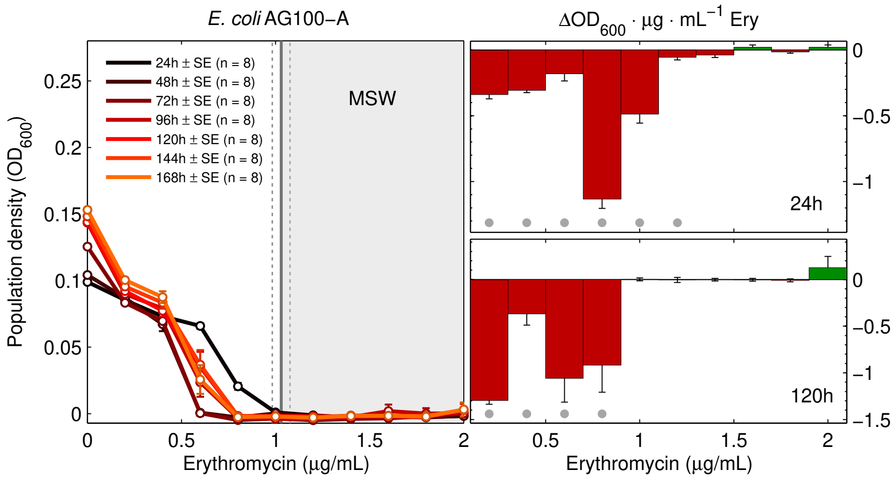

A)

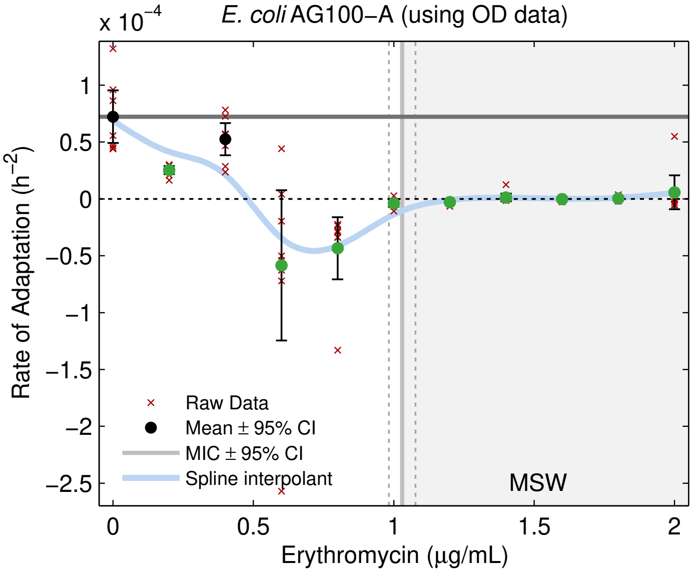

B)

Figure S3. The dose response of AG100A hardly changes during a 7-day erythromycin treatment. A) The leftmost subplot illustrates little daily change in the dose response data during treatment. The MIC and the predicted MSW (mutant selection window - shown with a 95% CI) measured after 24h are represented by the grey region and two dashed lines either side of the MIC. The effect on bacterial density of increments in drug concentration are shown in the two rightmost subplots after 24h and 120h of exposure to erythromycin (mean ± s.e., $n = 8$): all are negative, thus increased drug dose increases growth inhibition at all times here. Significant changes from a 2-sided t-test are denoted by a grey dot. **B)** Rates of adaptation at different drug concentrations determined using r_{auc}. The dashed line (with zero intercept) indicates where no detectable adaptation occurs, the thick horizontal grey line marks the rate of adaptation to the growth media as observed in the absence of erythromycin. Significant changes with respect to the latter are denoted by green dots based on two-sided t-tests (mean ± s.e., $n = 8$). Thus, AG100-A adapts significantly more quickly when not treated with erythromycin than if treated, say, near to its erythromycin MIC.

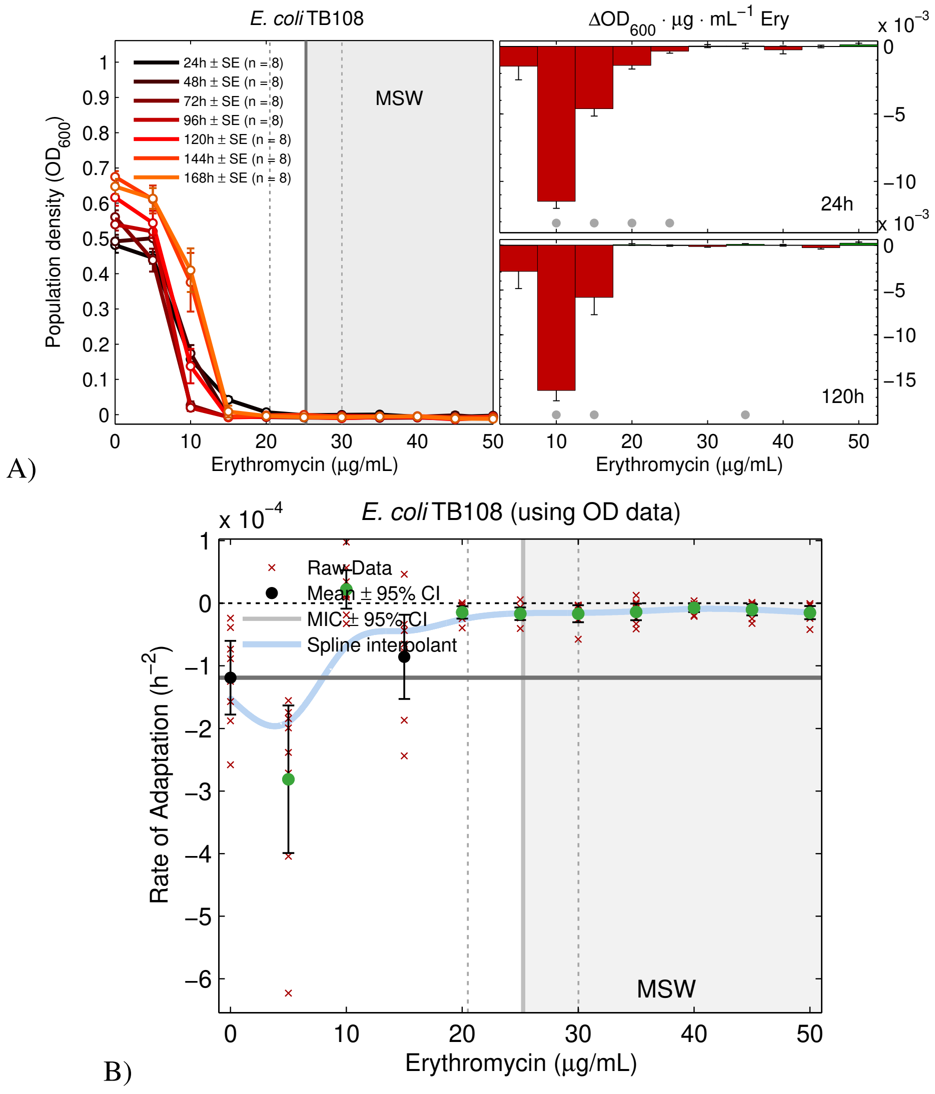

FIGURE S4. This is the analogy of Figure S3 for TB108.

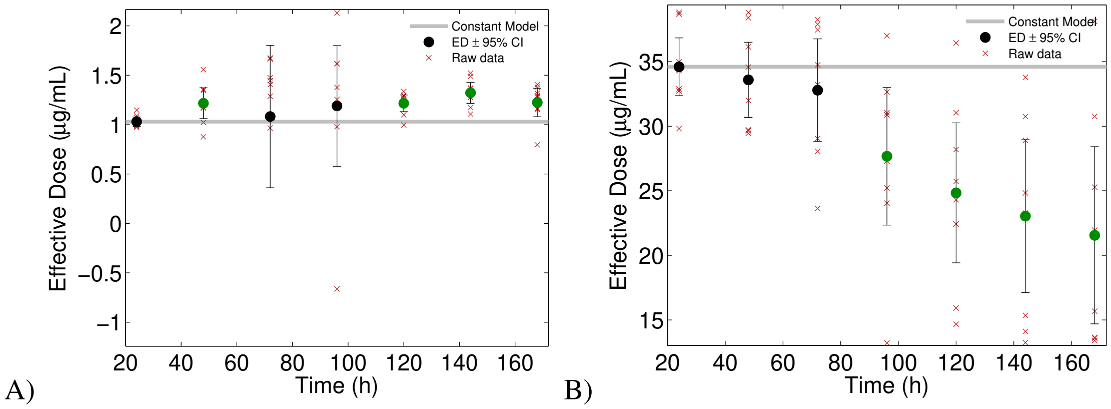

FIGURE S5. Dynamics of the effective antibiotic dose (EAD). A) The EAD does not change for AG100A when treated (see Methods). B) The EAD does change when eTB108 is treated, reducing in value by approximately 50%. Significant changes based on a 2-sided t-test with $p < 0.05$ are shown as a green dot, non-significant changes are shown as a black dot.

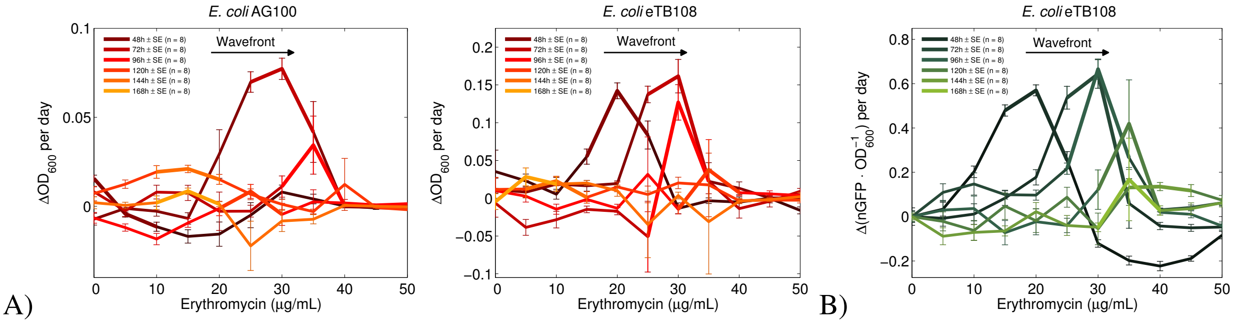

FIGURE S6. Daily changes in bacterial density at different drug concentrations for 3 strains. Beginning on day two, each line in A and B represents the difference in bacterial density (orange) or relative GFP fluorescence (green) between a given day and the preceding day's data. For strain eTB108, comparing data in A and B suggests a correlation between changes in bacterial density and efflux pumps per cell, where a proxy for the latter in B is the daily change in GFP units per OD signal. This correlation is affirmed and quantified in Figures S13 and S2.

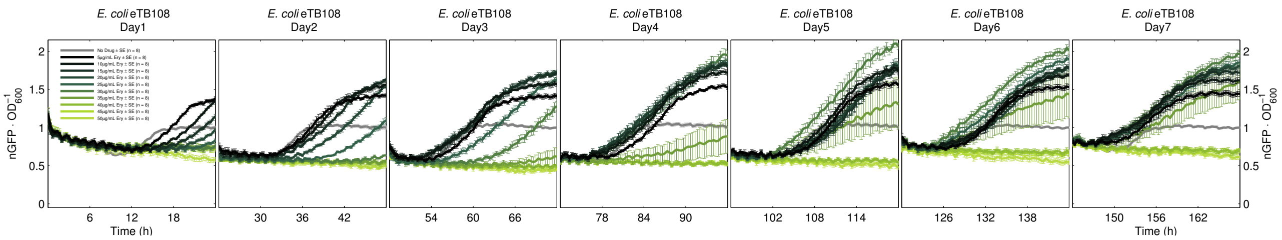

FIGURE S7. Dynamics of GFP per OD for eTB108 shown here are a proxy for mean AcrB-GFP per cell for 7 daily treatments. Note the within-season changes from lag to stationary phases at all of the antibiotic dosages used, note also the 'logistic shape' of each curve and the increasing AcrB-GFP levels through time each day. By the end of treatment only the very-highest dosages have not seen a rise in AcrB-GFP levels. The grey curve is the antibiotic-free control dataset for which AcrB-GFP levels remain similar, forming a logistic curve each day.

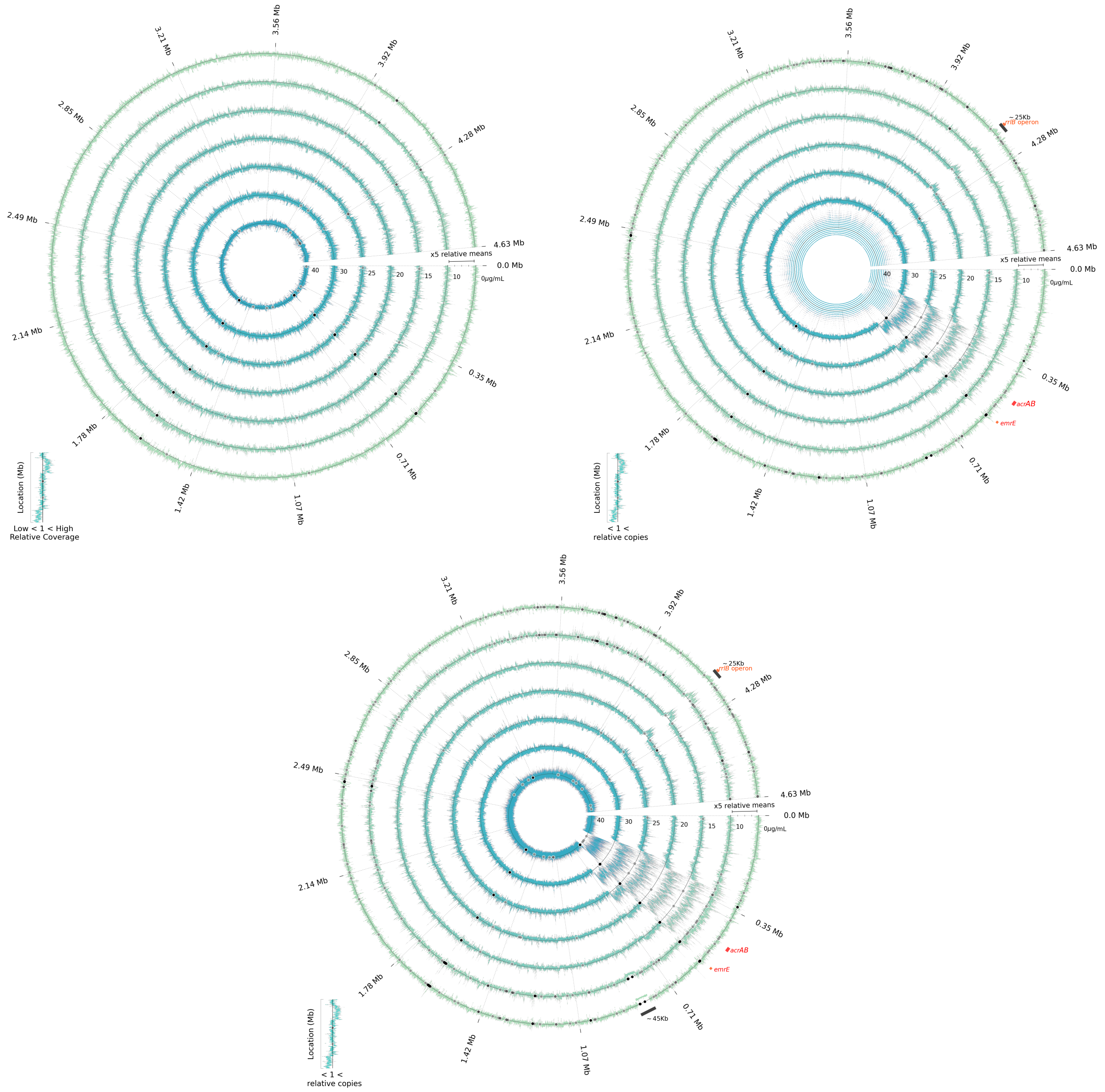

Figure S8. Relative coverage at different erythromycin doses (Replicate 1). Normalised Illumina coverage data of AG100 metagenomes treated at different erythromycin concentrations after 1 (top-left), 3 (top-right) and 5 (middle bottom - a repeat of Figure 4) days. Each outermost ring shows populations treated with no antibiotic, inner rings show data for increasing concentrations of drug (10-40µg/mL); Figures S9 and S10 show 2 more replicates. Larger values (visible spikes) represent genomic amplification, smaller values (visible dips) indicate gene deletions. SNPs are indicated by dots in the corresponding chromosomal location. The operon *acrAB* is highlighted in red between 480,553-485,707 bp. The loss of ~45Kb (804,268-849,700 bp) and the amplification of a ~25kb region between 4,164,724-4,189,435 bp, are highlighted in black (genes in this region are listed in Supplement 5).

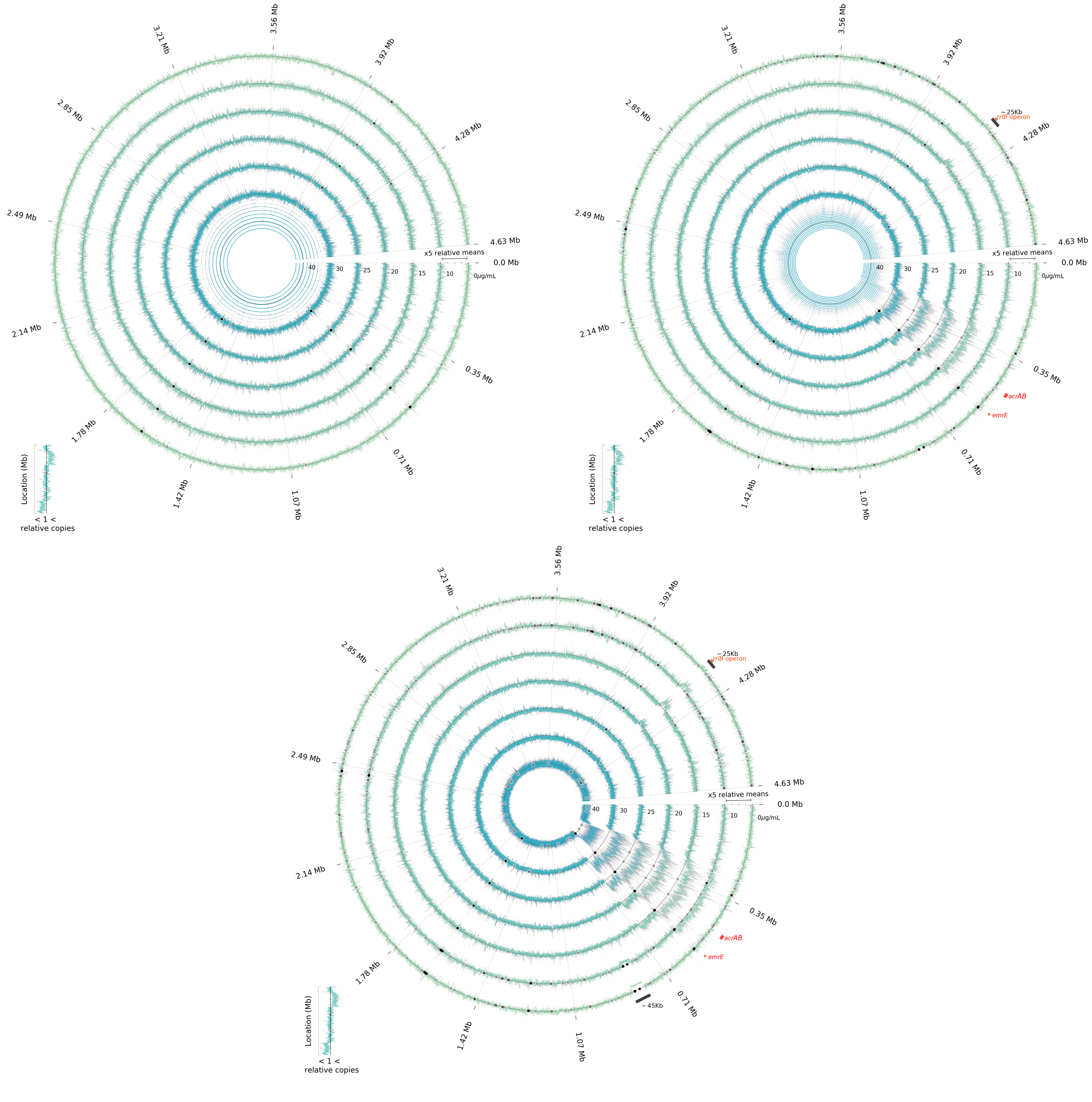

FIGURE S9. A technical replicate of Figure S8.

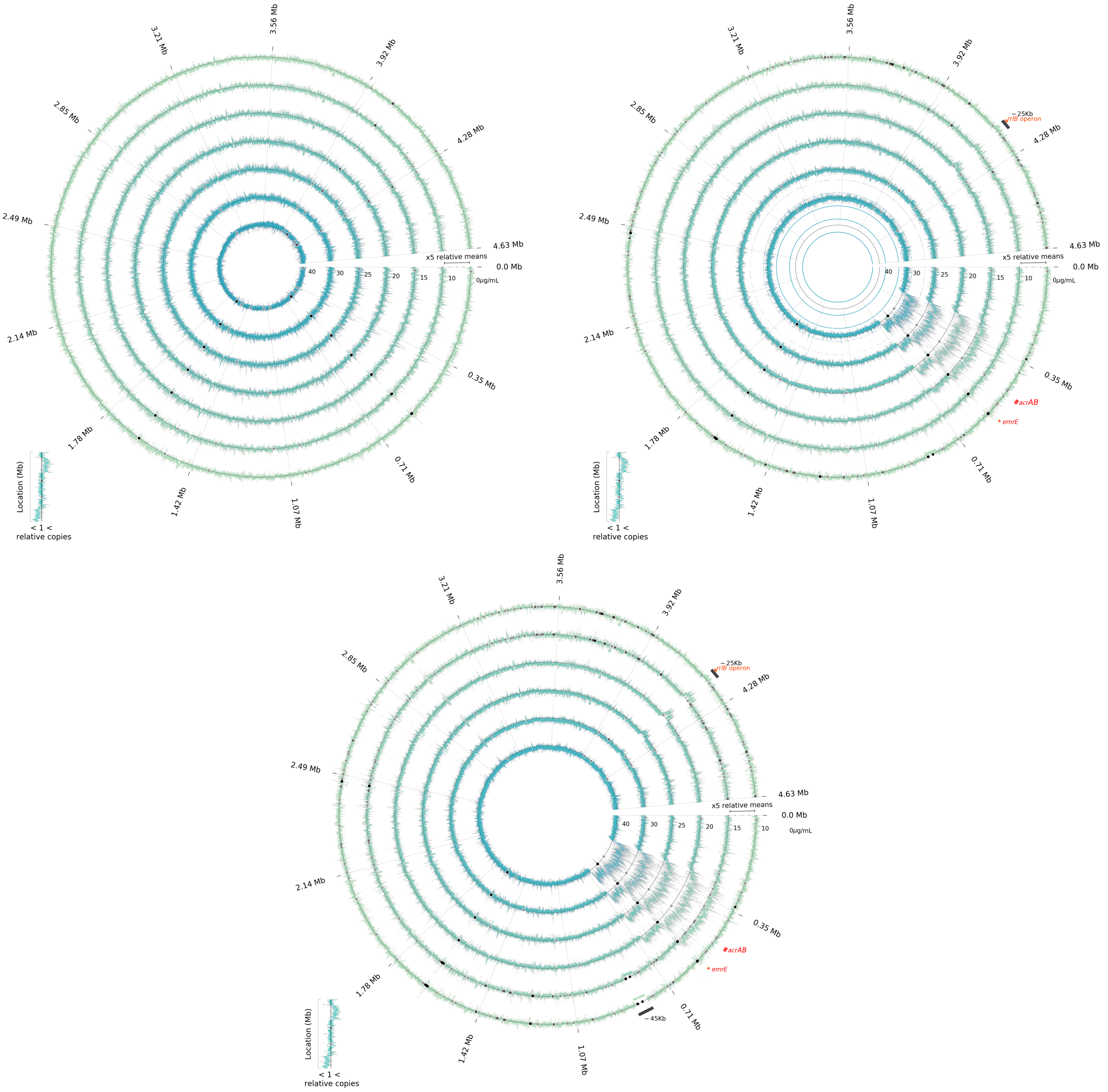

Figure S10. A technical replicate of Figure S8.

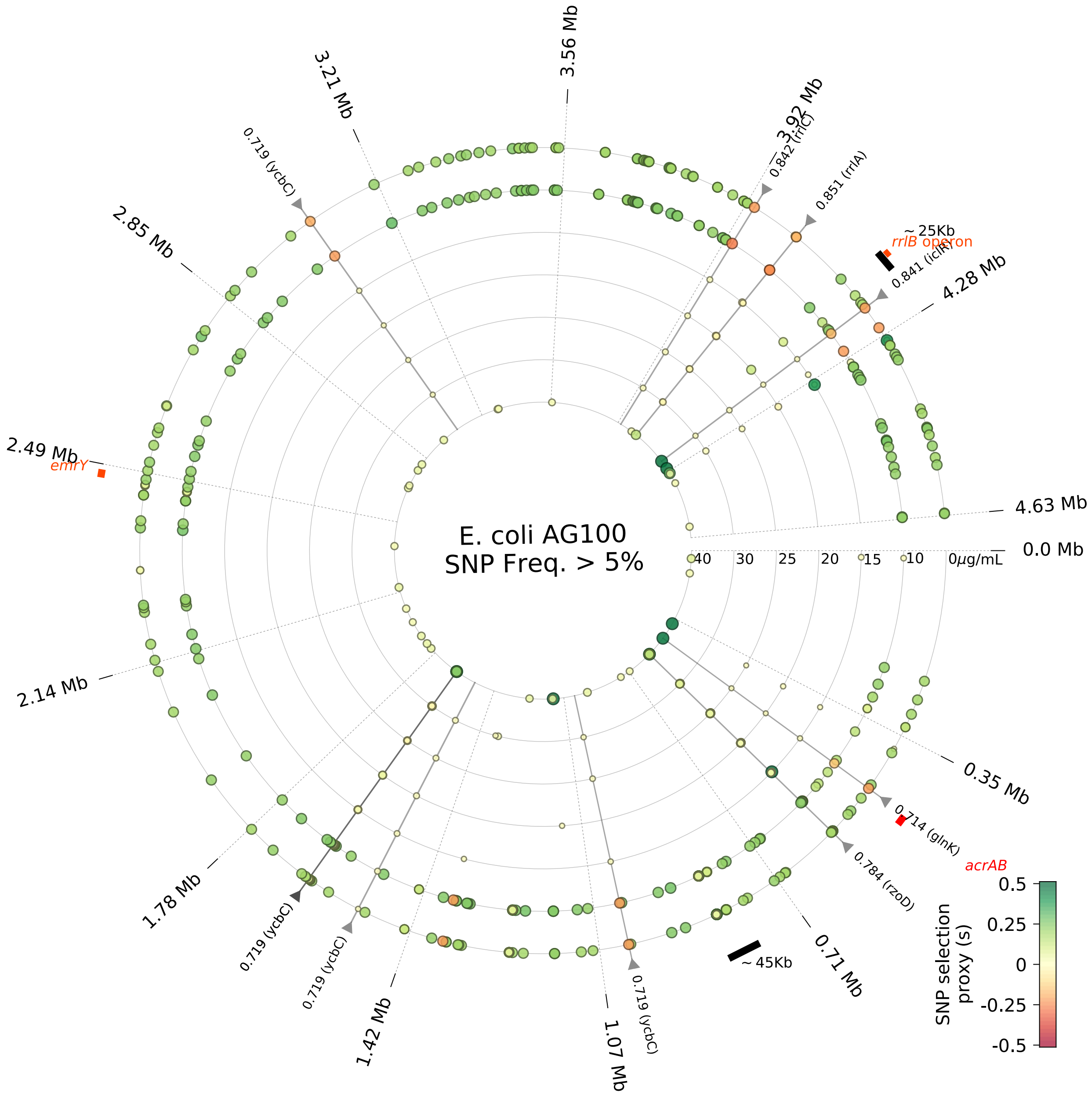

FIGURE S11. A technical replicate of Figure 4

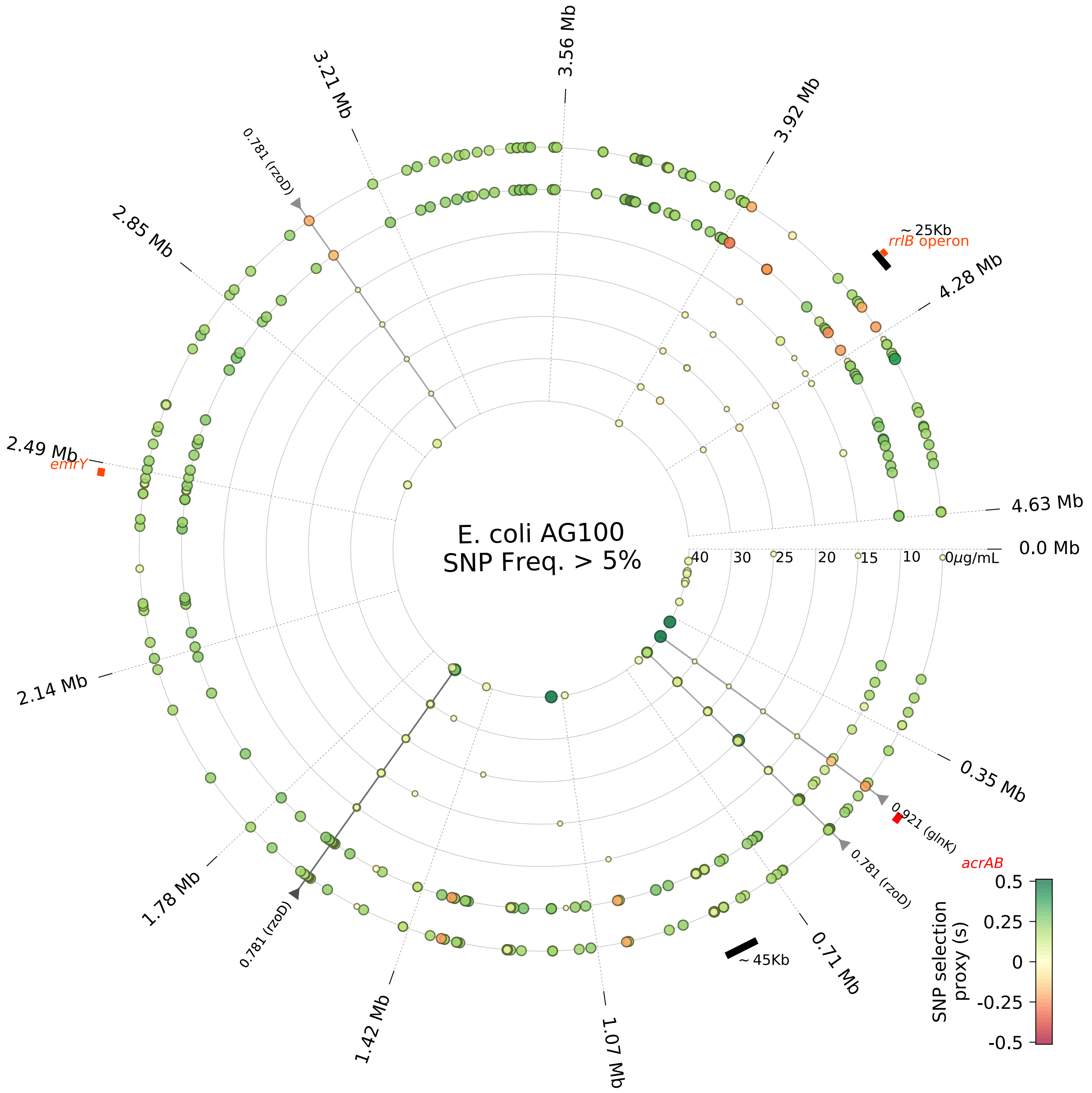

Figure S12. A technical replicate of Figure 4

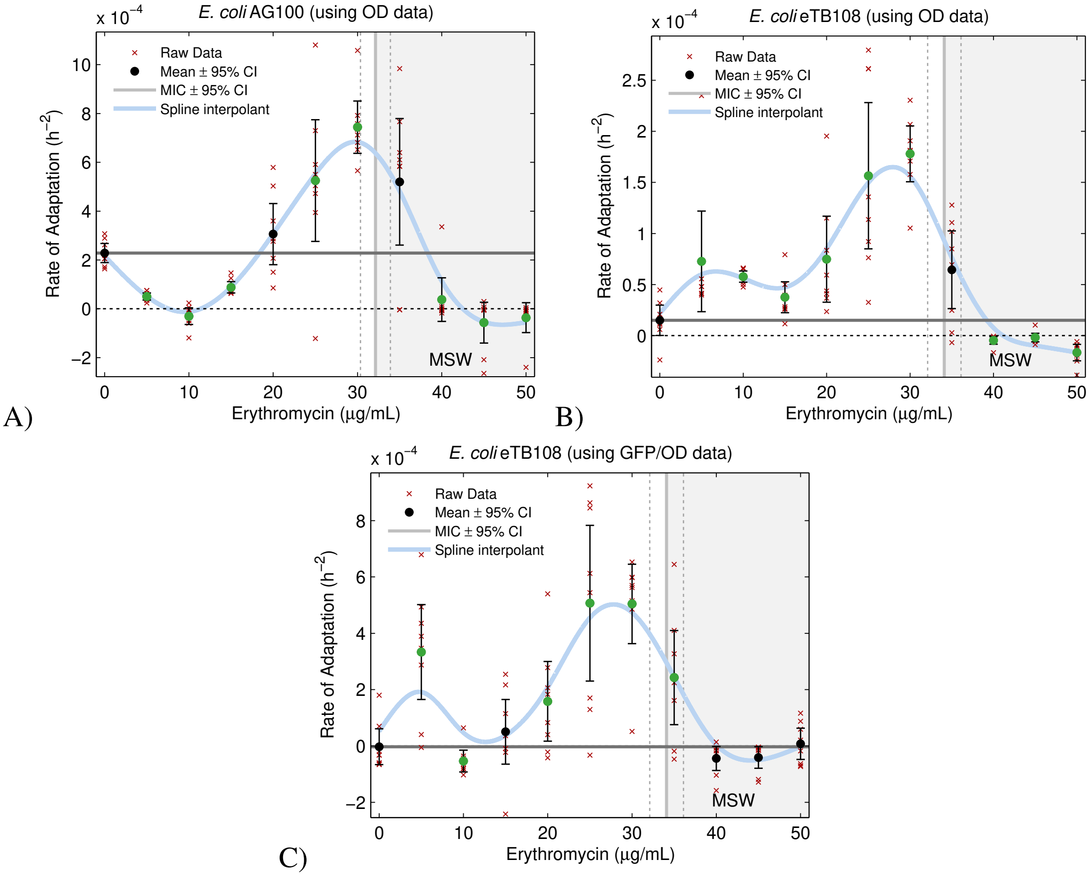

Figure S13. Population density rates of adaptation (defined using r_e, Methods) peak below the MIC. Rates of adaptation (ROA) determined using r_e (see Methods) vary with erythromycin concentration. A and B use population density (OD) data whereas C uses the mean relative abundance of AcrB (GFP per OD). The thick cyan line interpolates ROA data using a spine to guide the eye. The thick grey line is a baseline determined by propagation of the strains in liquid media without antibiotic and the dashed line denotes a ROA of zero. Significant differences with respect to the baseline are green dots based on two-sided t-tests (mean ± s.e. with $n = 8$).

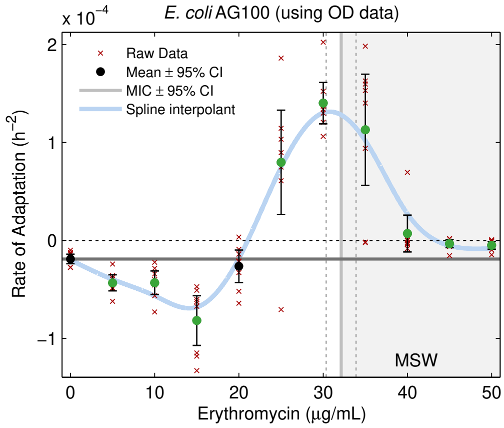

Figure S14. Rates of population density adaptation depend on drug concentration. Population density rates of adaptation (ROA) for strain AG100 measured using r_{auc} and applied to OD data. Like the analysis of ROA that uses r_e (Figure S13A) these vary with drug concentration and peak near the MIC.

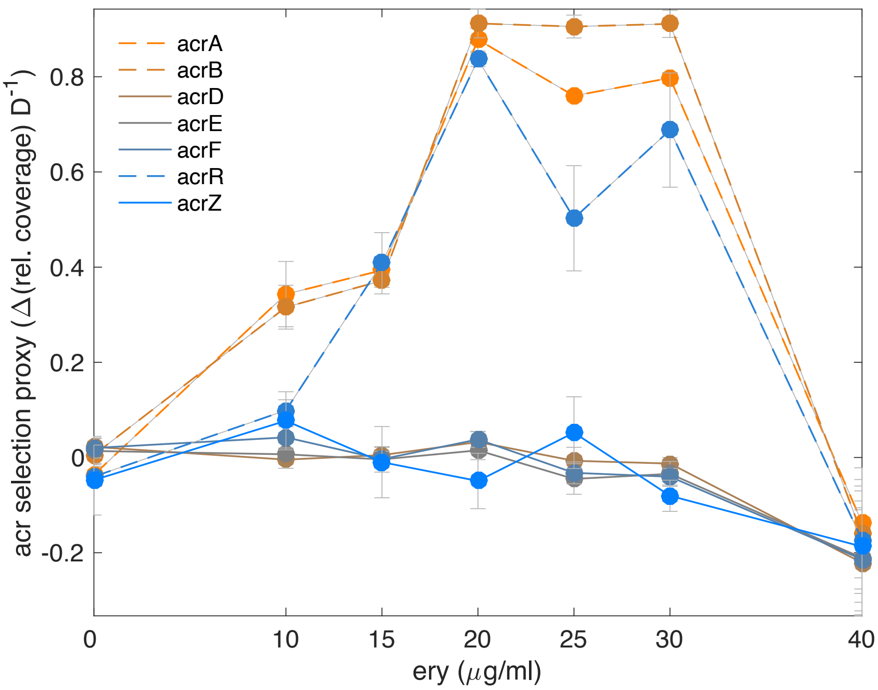

Figure S15. Not all efflux operons for which erythromycin is a substrate have an inverted-U geometry. Selection coefficients for amplifications of the operon *acrAB* are compared against members of the efflux operon *acrEF*. These data show the latter is maintained at constant levels in the AG100 genome throughout treatment and their selection proxy for amplifications is, as a result, close to zero for all dosages (see Methods).

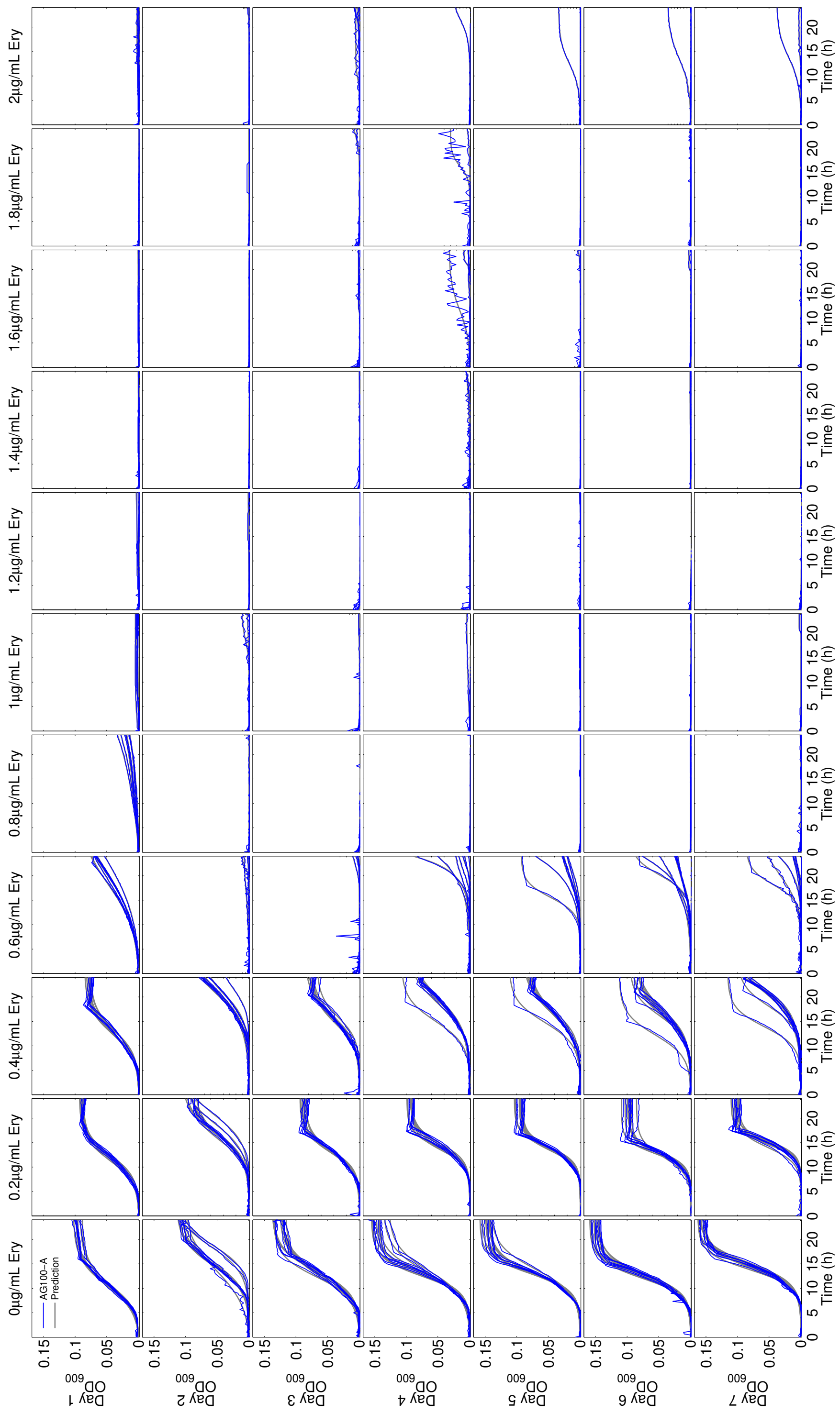

Figure S16. Raw OD data (blue) with logistic model fits (grey) for all treatments of AG100A. OD_{600nm} was read every 20min for 24h for 7 days. Each box shows 24h worth of data for 8 replicates where erythromycin doses are indicated.

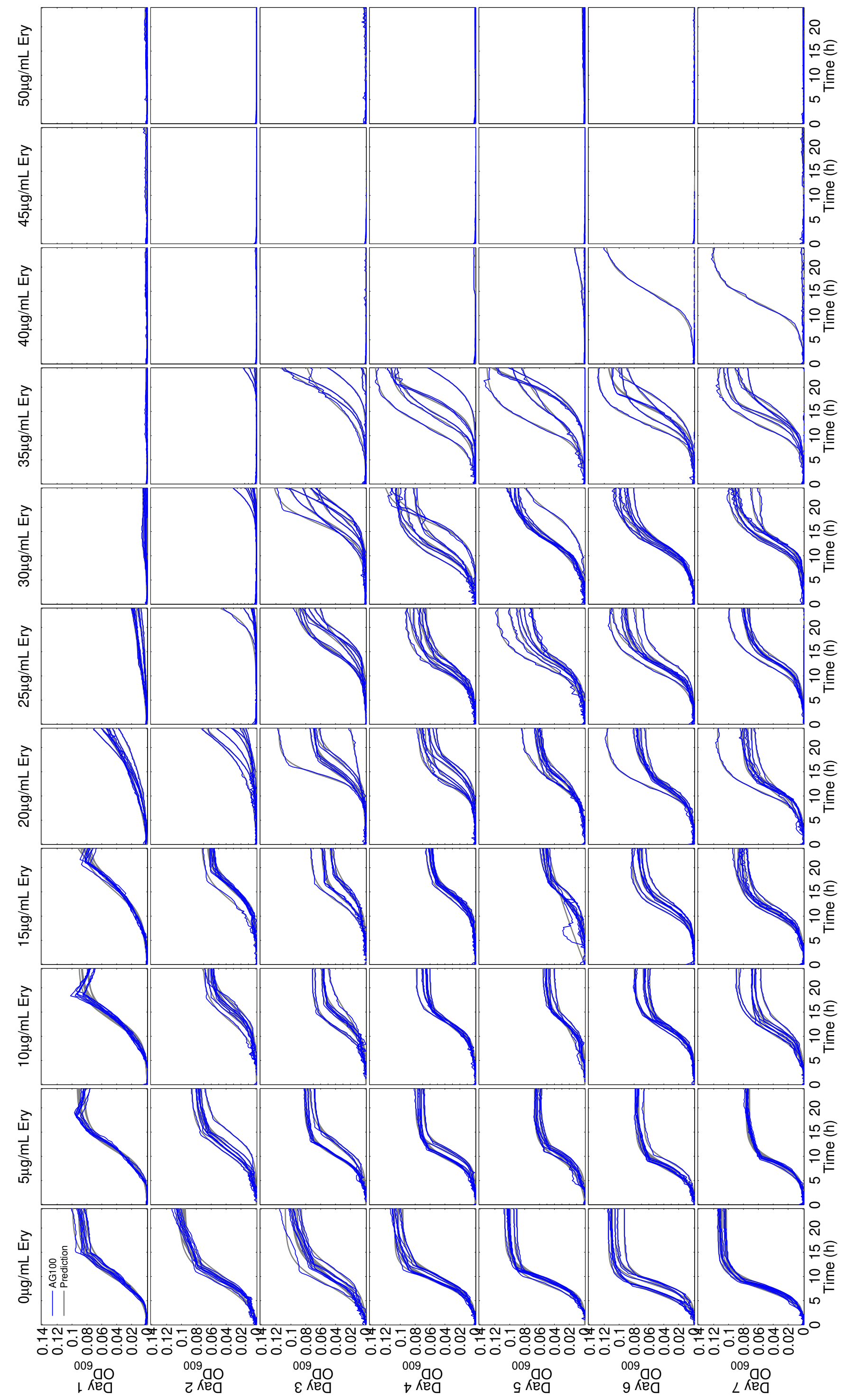

Figure S17. Analogous to Figure S16 but for AG100. The MIC is around $30\mu g/ml$ erythromycin (Ery) but population growth is observed in some replicates up to $40\mu g/ml$ following 5 days of treatment.

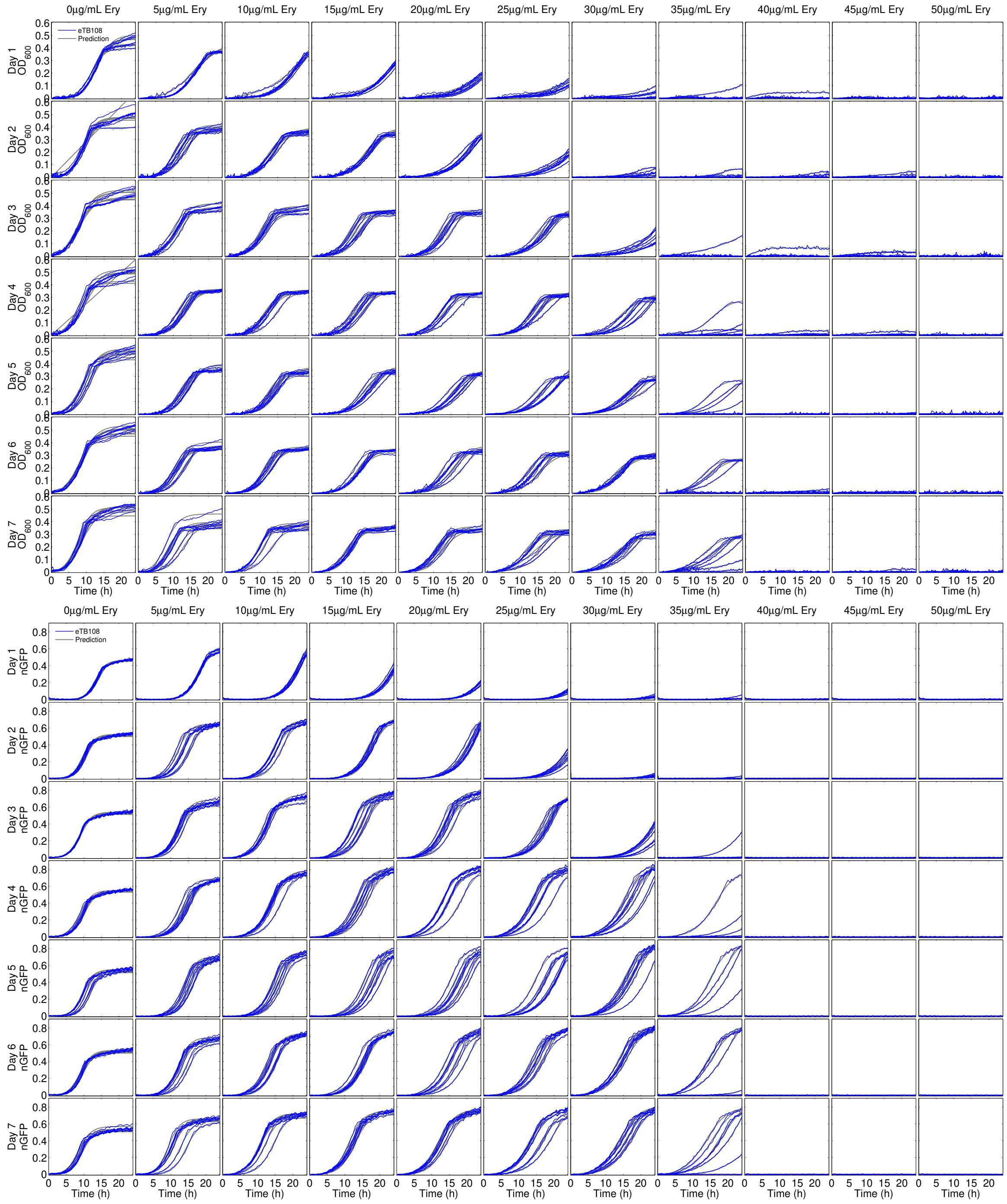

FIGURE S18. Raw OD (top) and GFP (bottom) data and associated model fits for eTB108. 24h population growth curves are shown for *E. coli* eTB108 based on optical density at 600nm (OD_{600}, top) and the normalised fluorescence (nGFP, bottom) were both measured every 20min for 7 days. Each box contains data for 8 replicates at the indicated doses erythromycin for days 1 to 7.

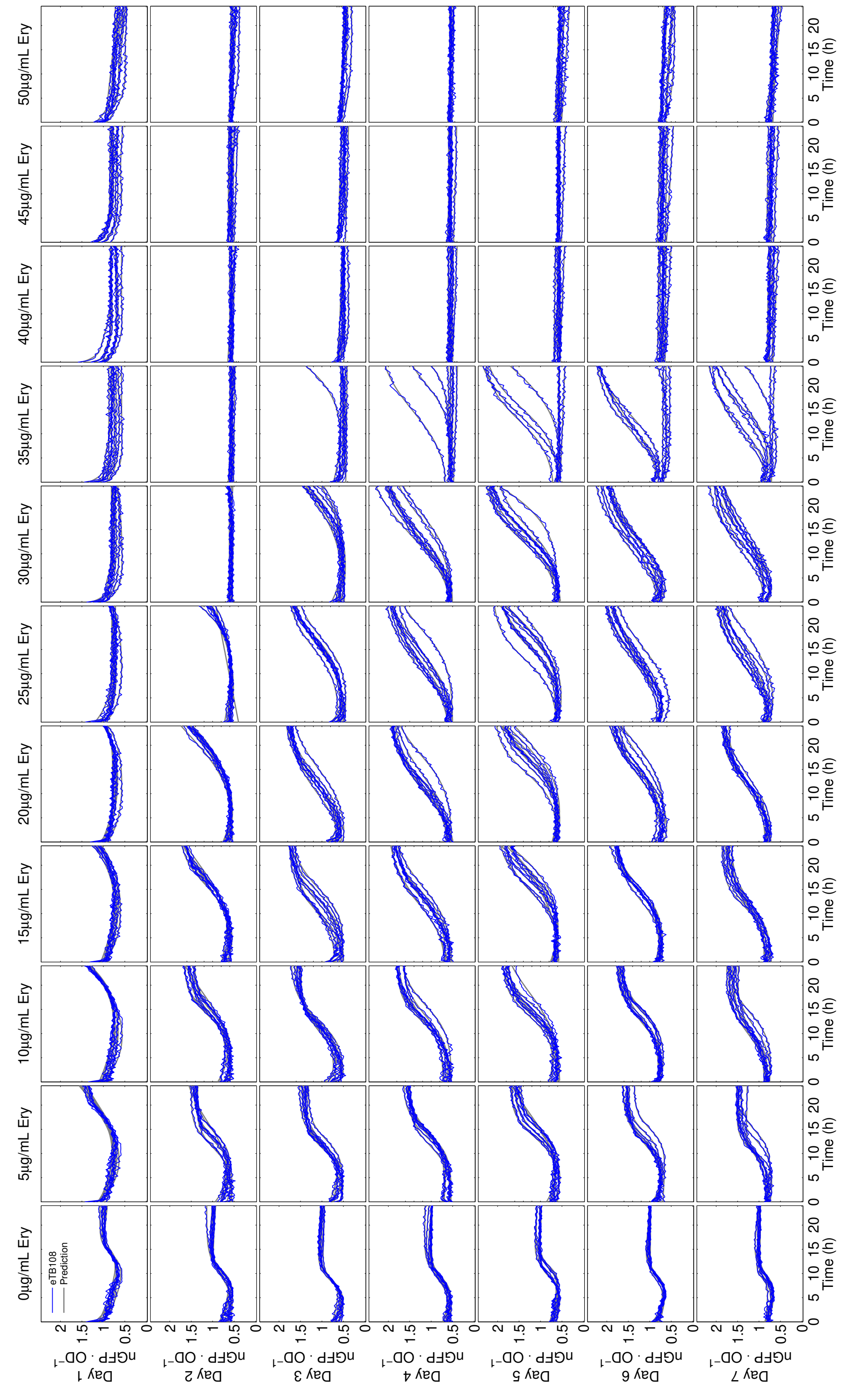

FIGURE S19. Relative fluorescence (GFP per OD) and associated model fits derived from Figure S18.

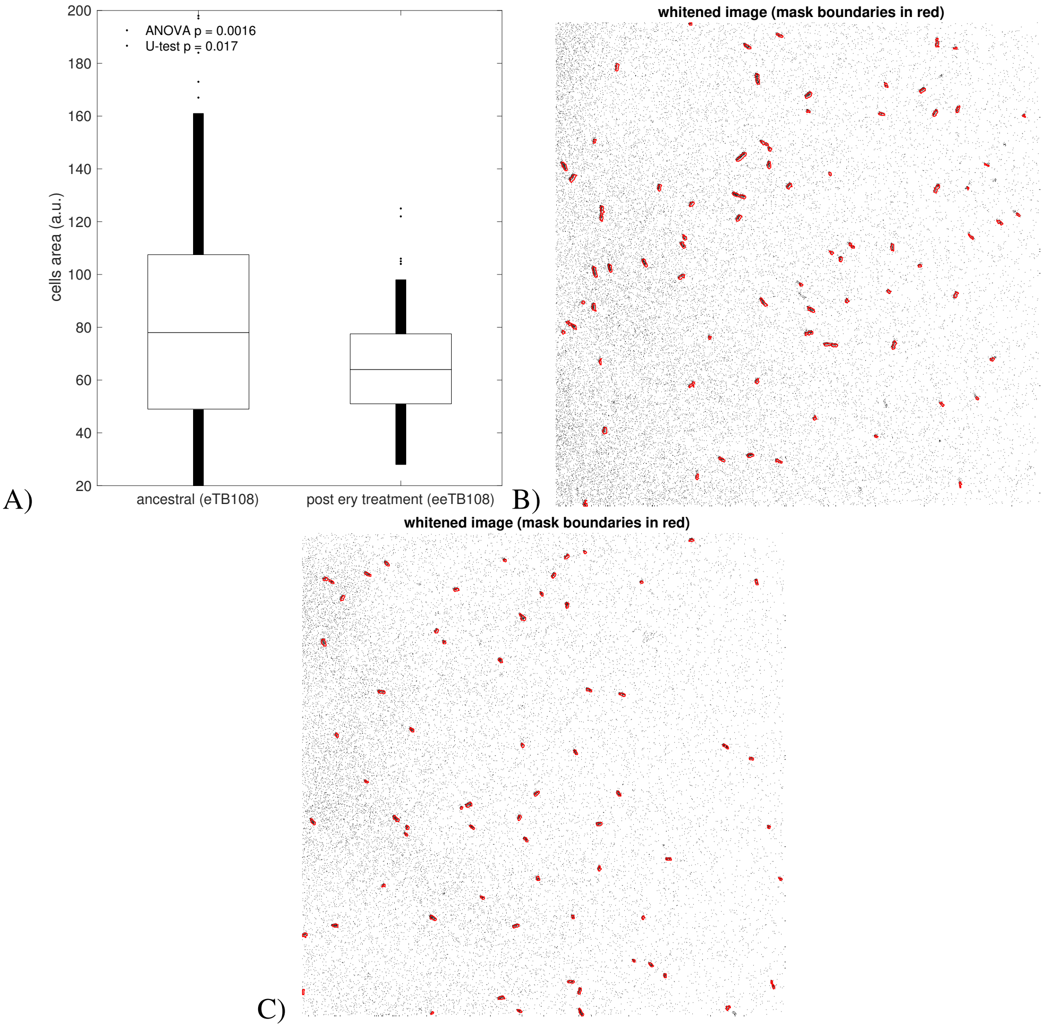

FIGURE S20. Cell size of eTB108 can decrease during erythromycin treatment. A) Anova to compare sizes of ancestral strain (eTB108) and its drug-treated descendant (eeTB108) isolated after the former was treated at sub-MIC erythromycin (Methods). Both were imaged following isolation in mid-exponential phase during which eeTB108 was exposed to erythromycin and eTB108 was not: the median size of eeTB108 is approximately 82% the area (in pixels) of eTB108 (respective medians 64 and 78, Interquartile ranges [51,77.8] and [48.5,107.8] pixels). B-C) Typical segmented images for the anova show *circa* 70 bacterial cells where B) is for eTB108 and C) shows eeTB108. Both show data analysis masks as red lines that mark the cells' edges.

A) 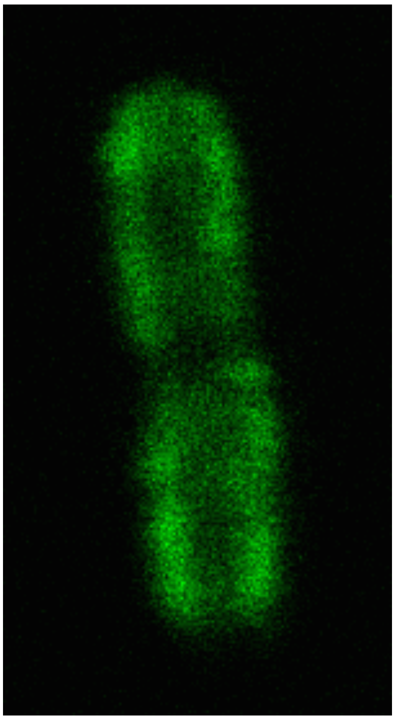 B) 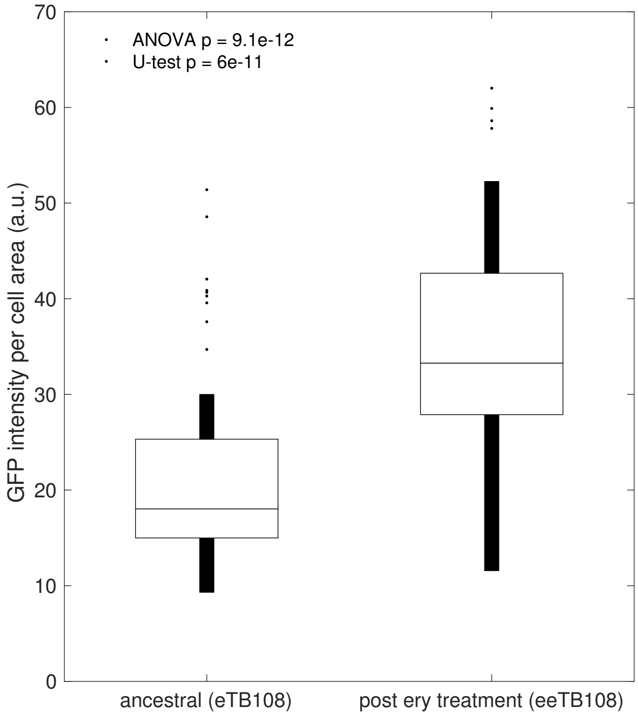

Figure S21. Imaging 2-fold increases in AcrB expression during erythromycin therapy. A) Fluorescence microscopy demonstrates the GFP-AcrB in TB108 has highest density towards the membrane of *E.coli*. B) Fluorescence microscope image data taken before (eTB108 data) and after (eeTB108 data) erythromycin treatment show eTB108 can double the expression of AcrB-GFP when treated at sub-MIC levels (strains described in Methods). GFP levels were measured using fluorescence microscopy and were quantified using edge detection in Matlab where algorithms report mean green channel pixel intensity per 2d projected cell area of approximately 70 cells pre- and post-treatment (respective eTB108 and eeTB108 medians are 18.0 and 33.3, interquartile ranges [14.9,25.4] and [27.6,42.7] relative pixel units, see Figure S20 for typical images). This represents a median change of *circa* 184%, corroborating analogous spectrophotometry data from the main text (e.g. the increases in GFP per OD reported in Figure 6A).

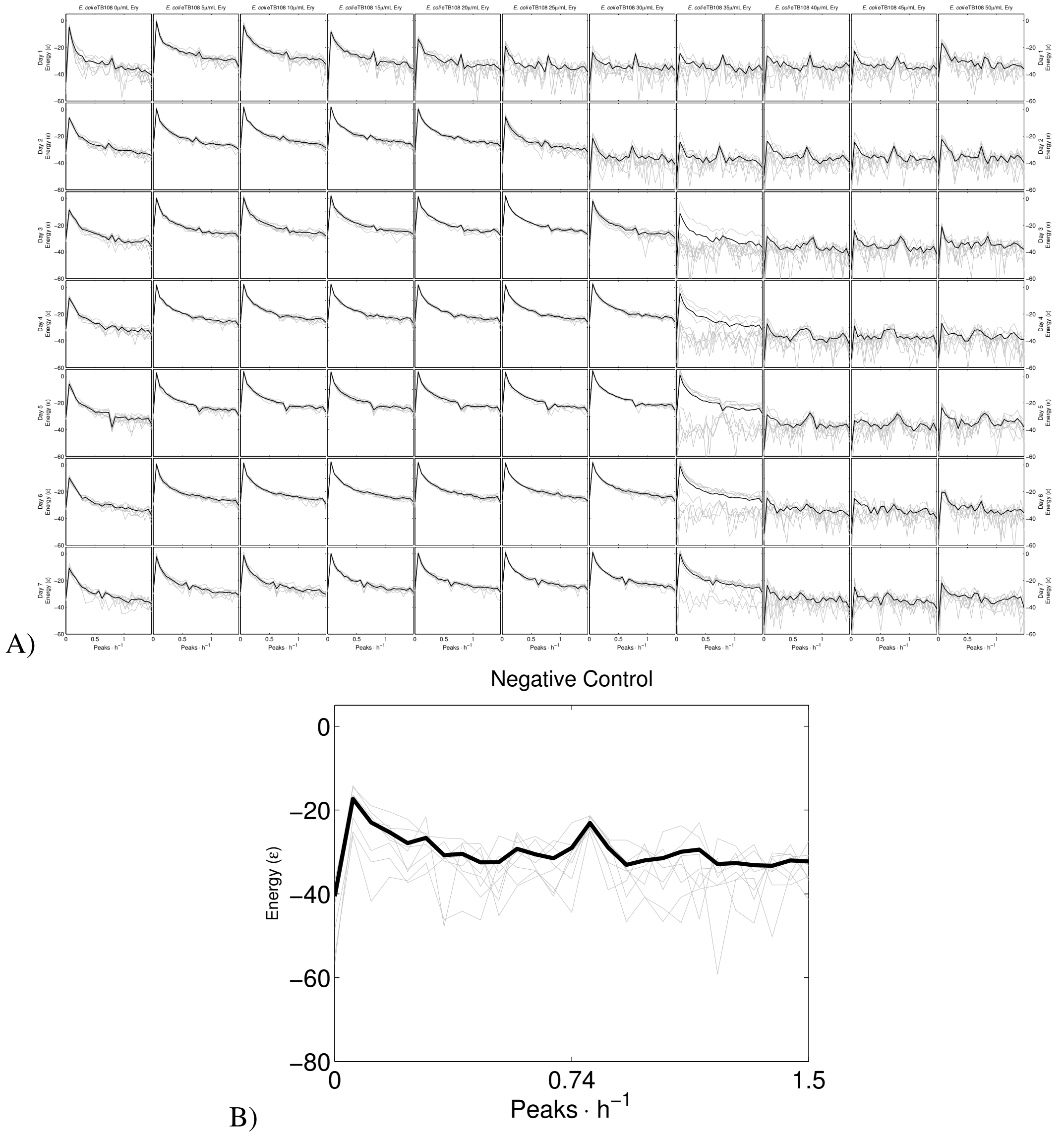

Figure S22. Fast Fourier transform (FFT) spectra for eTB108 GFP per OD data. **A)** The frequency of oscillations is shown on the x-axis and the ‘energy’ at each frequency on the y-axis. Days and concentrations of erythromycin are indicated and 2 features are apparent: a peak every ∼3/4 h and another every ∼10 h. The former corresponds to small oscillations observed in Figure 3D. **B)** The same information as A is shown but for non-inoculated microtitre plates and these also show a peak near 3/4h. We conclude that oscillations in GFP per OD data (also see Figure S7) do not have a biological basis but are a feature of the spectrophotometer device.

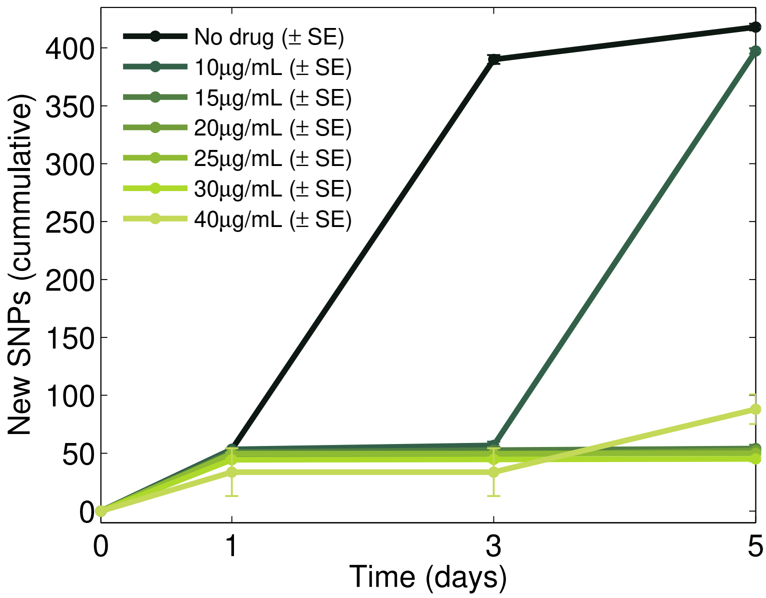

Figure S23. Cumulative novel SNPs per genome in each treatment on days 1, 3 and 5. Most SNPs (above 5% frequency, see Methods) were observed in the absence of erythromycin where population densities were highest.

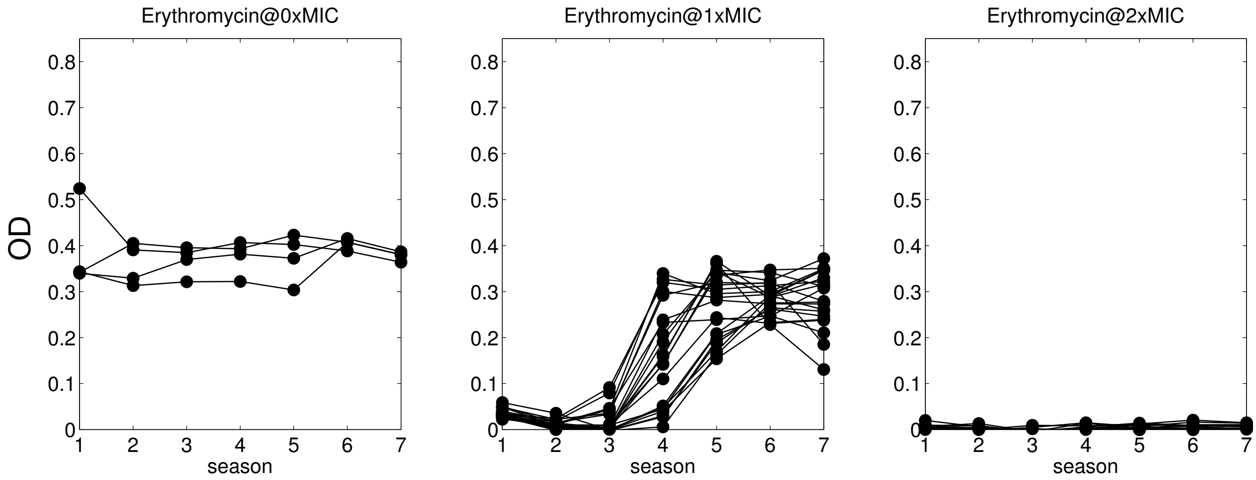

Figure S24. No population growth was detected at 2xMIC erythromycin. AG100 OD data for 7×24h 'seasons' of treatment at (left) 0xMIC, (middle) 1xMIC erythromycin and (right) 2xMIC (16 replicates when drug is present): no growth was detected at 2xMIC whereas OD increases towards no-drug levels in the 1xMIC treatments by season 7.

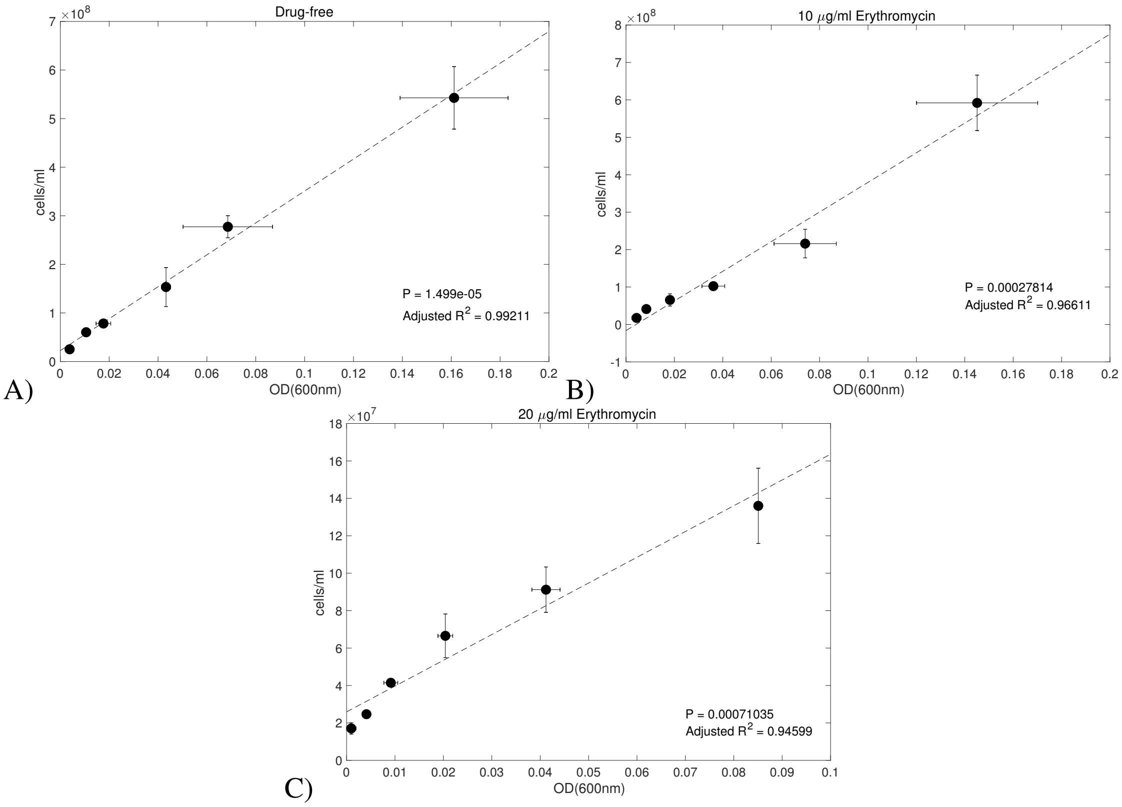

Figure S25. High correlations between OD and live cell counts (colony forming units - CFUs) were observed for eTB108 in the presence and absence of erythromycin. A) CFUs versus OD (600nm) in the absence of erythromycin show a high, positive correlation (see legend for statistics). B) The positive correlation between OD and CFU is maintained after introducing $10\mu g/ml$ of erythromycin into the culture medium. C) The positive correlation between OD and CFU is maintained after introducing $20\mu g/ml$ of erythromycin into the culture medium.

5 Contents of the 25Kb amplified region containing *rrlB* in Figure 4

Figure 4 highlights a 25Kb region (see the black band) containing the drug target *rrlB* that has increased coverage in populations exposed to erythromycin dosages around 10-$20\mu g/ml$. The *rrlB* datapoint in Figure 8A and also Figure 7A) confirm this region is amplified at rates that are commensurate with other *rrl* operons in the bacterial population. The only novel SNP detected in this 25Kb region resides in *argE* (acetylornithine deacetylase in the arginine biosynthetic process). The following genes are also amplified when *rrlB* is:

(1) transfer RNA genes *gltT*, *thrU*, *tyrU*, *glyT*, *thrT*;

(2) ribosomal genes: *rplK, rplA, rplJ, rplL*;

(3) RNA-polymerase: *rpoB* (beta subunit), *rpoC* (beta prime subunit);

(4) putative gene (yjaZ): adaptation to atypical conditions (heat shock protein);

(5) sRNA *sroH* of unknown function;

(6) cell wall (peptidoglycan) synthesis *murB*;

(7) *coA* (coenzyme A), *birA* (biotin synthesis), *thiH* (thiamin synthesis), *thiS* (sulphur carrier).

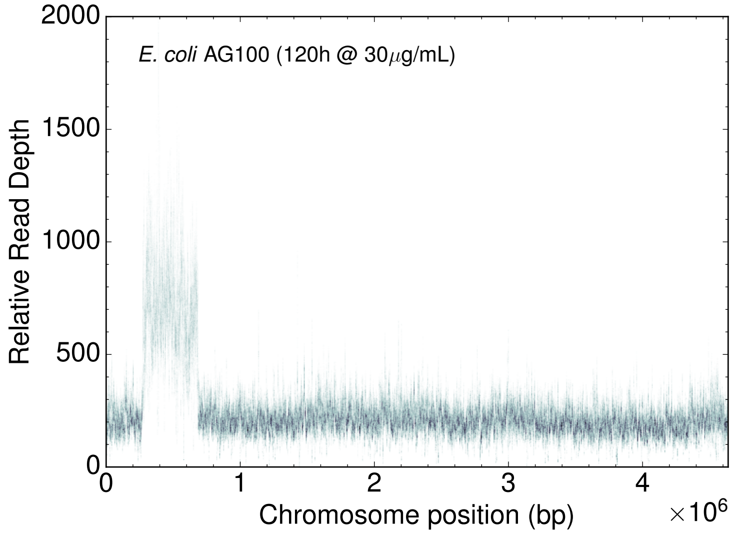

FIGURE S26. An exemplar plot of raw coverage data for a treated AG100 population. Mean (Illumina) coverage across the genome started just above 200 when treatment began. These maintained a level around (and over 200) throughout treatment for most of the genome but an amplified region is significantly above twice that by the end of treatment; these raw coverage data are for DNA data extracted after 120h of treatment at $30\mu g/ml$ erythromycin.

6 Supplementary Tables

The following 6 tables show novel SNPs detected above 5% frequency in their respective populations sampled from each of the different antibiotic treatments that are also not present in any of the drug-free control treatment replicates. The value of s_j is a selection proxy determined from the frequency dynamics of each SNP (Methods), where the s-coefficient is determined from the logistic model (5) after fitting it to frequency data, j denotes replicate 1, 2 or 3.

1.

base pair	gene	s_1	s_2	s_3	dose	annotation
1,123,847	*putP*	-	-	-0.001	10	sodium/proline symporter

2.

base pair	gene	s_1	s_2	s_3	dose	annotation
384,896	*tauA*	-0.001	-	-	15	taurine transporter subunit - sulfur metabolic process
4,168,030*	*rrlB*	0.11	0.11	0.11	15	23S ribosomal RNA of rrlB operon

3.

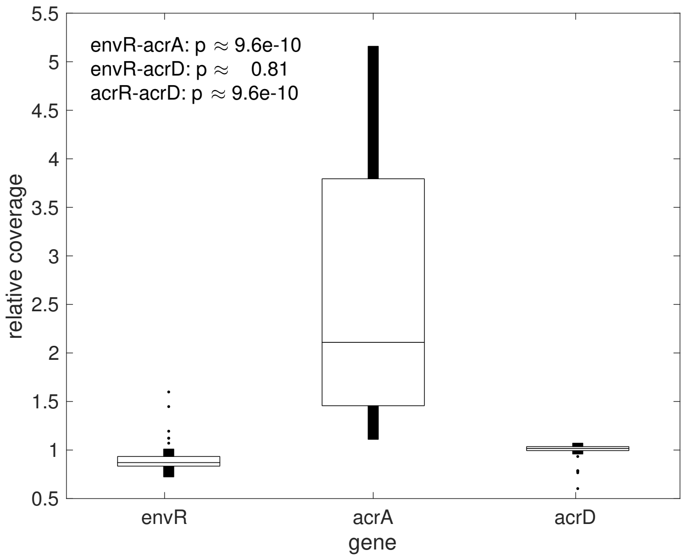

Figure S27. The envR gene is not amplified during treatment. Pairwise comparisons of relative coverage data collated across all times for the genes *acrA*, *acrD* and *envR* are indicated on the y-axis to show *acrD* and *envR* are not amplified: the relative coverage is approximately unity at all times at all dosages. This is not the case for *acrA* which is amplified. Matlab's `multcompare` and `boxplot` commands generated pairwise *p* values to test for significant differences in data, as indicated in the legend.

base pair	gene	s_1	s_2	s_3	dose	annotation
1,123,847	*putP*	-	0.002	-0.002	20	sodium/proline symporter
1,352,810	*yciQ*	0.01	-	-	20	putative inner membrane protein
2,761,715	*yfjL*	0.012	-	-	20	CP4-57 prophage; putative protein
4,168,030	*rrlB*	0.15	0.14	-	20	23S ribosomal RNA of rrlB operon

4.

base pair	gene	s_1	s_2	s_3	dose	annotation
384,896	*tauA*	-0.001	-	-	25	taurine transporter subunit - sulfur metabolism
4,168,030	*rrlB*	0.07	-	-	25	23S ribosomal RNA of rrlB operon

5.

base pair	gene	s_1	s_2	s_3	dose	annotation
1,352,810	*yciQ*	-	0.0189	-	30	putative inner membrane protein

base pair	gene	s_1	s_2	s_3	dose	annotation
40,068	*caiA*	-	0.0741	-	40	crotonobetaine reductase subunit II, FAD-binding / probable carnitine operon oxidoreductase - glyoxylate cycle
60,661	*rluA*	-	-	0.0588	40	23S rRNA pseudouridine(746), tRNA pseudouridine(32) synthase
114,001	*guaC*	-	-	0.0667	40	GMP reductase - purine ribonucleotide biosynthesis
114,678	*guaC*	-	0.0400	-	40	GMP reductase - purine ribonucleotide biosynthetis
125,303	*aceE*	-	-	0.0667	40	pyruvate dehydrogenase, decarboxylase component E1, thiamin-binding
164,499	*hrpB*	-	-	0.0625	40	putative ATP-dependent helicase - DNA replication
176,505	*clcA*	-	-	0.0238	40	H(+)/Cl(-) exchange transporter - putative channel
272,541	*ykfC*	-	-	0.0500	40	pseudogene / CP4-6 prophage
384,896	*tauA*	0.5000	-	0.5000	40	taurine transporter subunit - sulfur metabolism
505,711	*ushA*	0.0213	-	-	40	bifunctional UDP-sugar hydrolase/5'-nucleotidase - nucleotide-sugar biosynthetisis
635,476	*dsbG*	-	-	0.0500	40	thiol:disulfide interchange protein
709,167	*ybfF*	-	0.0278	-	40	acyl-CoA esterase
762,513	*mngR*	-	0.0238	-	40	DNA-binding transcription repressor, fatty-acyl-binding, regulates succinylCoA synthetase - TCA cycle
948,499	*ybjS*	-	0.0588	-	40	putative NAD(P)H-binding oxidoreductase with Rossmann-fold domain
1,054,974	*pqiA*	-	-	0.0278	40	paraquat-inducible membrane protein A
1,116,859	*rutR*	0.0476	-	-	40	DNA-binding transcriptional repressor for rut operon
1,123,847*	*putP*	0.5000	0.5000	0.5000	40	proline:sodium symporter - proline biosynthetic process
1,127,111	*efeB*	-	0.0588	-	40	deferrrochelatase, periplasmic
1,185,116	*rne*	0.1250	-	-	40	ribonucleaseE: endoribonuclease/RNA-degradosome binding protein, mRNA turnover, maturation 5S RNA
1,243,589	*ymgA*	-	0.0417	-	40	RcsB connector protein for regulation of biofilm
1,291,949	*hemA*	0.0588	-	-	40	glutamyl tRNA reductase glutamyl-tRNA reductase
1,458,461	*paaA*	-	-	0.0625	40	ring 1,2-phenylacetyl-CoA epoxidase subunit carbohydrate catabolic process
1,468,707	*paaY*	0.0333	-	-	40	putative hexapeptide repeat acetyltransferase
1,656,949	*rspB*	-	-	0.0588	40	putative oxidoreductase, Zn-dependent and NAD(P)-binding, starvation sensing protein
1,813,675	*yniC*	-	0.0556	-	40	2-deoxyglucose-6-P phosphatase, putative phosphatase
1,845,698	*ynjH*	-	0.0556	-	40	conserved protein, DUF1496 family
1,893,553	*fadD*	-	0.0526	-	40	acyl-CoA synthetase (long-chain-fatty-acid–CoA ligase), fatty acid oxidation
1,975,479	*tar*	-	0.0294	-	40	methyl-accepting chemotaxis protein II, aspartate sensor receptor
2,051,293	*yeeJ*	-	0.0400	-	40	putative adhesin, regulation of transcription
2,164,624	*mdtD*	-	0.0625	-	40	putative arabinose efflux transporter, putative transport protein - peptidoglycan-based cell wall
2,374,605	*arnT*	-	0.0357	-	40	4-amino-4-deoxy-L-arabinose transferase, (lipid A modification) - response to stress
2,678,419	*yphG*	-	0.0556	-	40	hypothetical protein
2,687,345	*glmY*	-	-	0.0667	40	sRNA activator of glmS mRNA, glmZ processing antagonist
2,687,347	*glmY*	-	-	0.0667	40	sRNA activator of glmS mRNA, glmZ processing antagonist
2,691,546	*purL*	-	0.0333	-	40	phosphoribosylformyl-glycineamide synthetase, purine nucleotide biosynthetis
2,746,313	*yfjD*	0.0667	-	-	40	inner membrane protein, UPF0053 family
2,778,914	*ypjA*	-	0.0455	-	40	adhesin-like autotransporter, putative ATP-binding component of a transport system
2,814,924	*serV*	-	0.0526	-	40	tRNA-Ser, anticodon: GCU
2,945,400	*amiC*	-	-	0.1053	40	N-acetylmuramoyl-L-alanine amidase - peptidoglycan biosynthesis
2,980,625	*kduI*	-	0.0500	-	40	4-deoxy-L-threo-5-hexosulose-uronate ketol-isomerase, pectin degrading 5-keto 4-deoxyuronate isomerase homolog
2,985,761	*yqeJ*	0.0323	-	-	40	hypothetical protein
3,118,194	*glcB*	0.1333	-	-	40	malate synthase G - carbohydrate catabolic process
3,209,867	*rpoD*	0.0500	-	-	40	RNA polymerase, sigma 70 (sigma D) factor, regulation of proteins induced at high temperatures
3,209,879	*rpoD*	0.0500	-	-	40	RNA polymerase, sigma 70 (sigma D) factor
3,209,882	*rpoD*	0.0455	-	-	40	RNA polymerase, sigma 70 (sigma D) factor
3,209,885	*rpoD*	0.0476	-	-	40	RNA polymerase, sigma 70 (sigma D) factor
3,209,889	*rpoD*	0.0435	-	-	40	RNA polymerase, sigma 70 (sigma D) factor
3,295,908	*yhbO*	-	0.0357	-	40	stress-resistance protein
3,300,675	*yhbW*	-	0.0333	-	40	putative enzyme
3,573,233	*gntU*	-	0.0238	-	40	gluconate transporter, split gene, low-affinity gluconate transport permease protein
3,673,699	*yhjG*	0.0625	-	-	40	Inner membrane protein, AsmA family
3,943,468	*trpT*	-	-	0.0312	40	tRNA-Trp anticodon: CCA
3,964,629	*wecA*	0.0526	-	-	40	UDP-GlcNAc:undecaprenylphosphate GlcNAc-1-phosphate transferase, surface antigen activity, host antigen biosynthesis
4,005,398	*rhtB*	-	0.0294	-	40	homoserine, homoserine lactone and S-methyl-methionine efflux pump, L-serine biosynthesis
4,183,197	*rpoC*	0.0400	-	-	40	RNA polymerase, beta prime subunit
4,296,346	*mdtP*	-	0.0526	-	40	outer membrane factor of efflux pump, putative enzyme - peptidoglycan-based cell wall
4,350,148	*lysU*	-	0.0238	-	40	lysine tRNA synthetase, inducible, heat shock protein - tRNA aminoacylation for protein translation
4,520,132	*yjhG*	0.0312	-	-	40	KpLE2 phage-like element; predicted dehydratase
4,582,359	*hsdR*	-	0.0370	-	40	endonuclease R Type I restriction enzyme, endonuclease R - DNA catabolic process
4,586,212	*yjiY*	0.0556	-	-	40	putative inner membrane protein, putative carbon starvation protein
